# A transistor-based point-of-care assay with lipid-capped sensory interface for clinical profiling of cardiovascular diseases

**DOI:** 10.1093/nsr/nwag156

**Published:** 2026-03-11

**Authors:** Duo Chen, Nan Song, Yun Zhang, Fuding Guo, Liping Zhou, Cunlan Guo, Xidong Duan, Dacheng Wei, Weihong Tan, Lilei Yu, Yanbing Yang, Quan Yuan

**Affiliations:** College of Chemistry and Molecular Sciences, Key Laboratory of Biomedical Polymers of Ministry of Education, Department of Cardiology, Institute of Molecular Medicine, Renmin Hospital of Wuhan University, State Key Laboratory of Metabolism and Regulation in Complex Organisms, Taikang Center for Life and Medical Sciences, Hubei Key Laboratory of Autonomic Nervous System Modulation, Cardiac Autonomic Nervous System Research Center of Wuhan University, Wuhan University, Wuhan 430072, China; College of Chemistry and Molecular Sciences, Key Laboratory of Biomedical Polymers of Ministry of Education, Department of Cardiology, Institute of Molecular Medicine, Renmin Hospital of Wuhan University, State Key Laboratory of Metabolism and Regulation in Complex Organisms, Taikang Center for Life and Medical Sciences, Hubei Key Laboratory of Autonomic Nervous System Modulation, Cardiac Autonomic Nervous System Research Center of Wuhan University, Wuhan University, Wuhan 430072, China; College of Chemistry and Molecular Sciences, Key Laboratory of Biomedical Polymers of Ministry of Education, Department of Cardiology, Institute of Molecular Medicine, Renmin Hospital of Wuhan University, State Key Laboratory of Metabolism and Regulation in Complex Organisms, Taikang Center for Life and Medical Sciences, Hubei Key Laboratory of Autonomic Nervous System Modulation, Cardiac Autonomic Nervous System Research Center of Wuhan University, Wuhan University, Wuhan 430072, China; College of Chemistry and Molecular Sciences, Key Laboratory of Biomedical Polymers of Ministry of Education, Department of Cardiology, Institute of Molecular Medicine, Renmin Hospital of Wuhan University, State Key Laboratory of Metabolism and Regulation in Complex Organisms, Taikang Center for Life and Medical Sciences, Hubei Key Laboratory of Autonomic Nervous System Modulation, Cardiac Autonomic Nervous System Research Center of Wuhan University, Wuhan University, Wuhan 430072, China; Key Laboratory of Cardiovascular Disease of Yunnan Province, Clinical Medicine Center for Cardiovascular Disease of Yunnan Province, Department of Cardiology, Yan’an Affiliated Hospital of Kunming Medical University, Kunming 650000, China; College of Chemistry and Molecular Sciences, Key Laboratory of Biomedical Polymers of Ministry of Education, Department of Cardiology, Institute of Molecular Medicine, Renmin Hospital of Wuhan University, State Key Laboratory of Metabolism and Regulation in Complex Organisms, Taikang Center for Life and Medical Sciences, Hubei Key Laboratory of Autonomic Nervous System Modulation, Cardiac Autonomic Nervous System Research Center of Wuhan University, Wuhan University, Wuhan 430072, China; College of Chemistry and Molecular Sciences, Key Laboratory of Biomedical Polymers of Ministry of Education, Department of Cardiology, Institute of Molecular Medicine, Renmin Hospital of Wuhan University, State Key Laboratory of Metabolism and Regulation in Complex Organisms, Taikang Center for Life and Medical Sciences, Hubei Key Laboratory of Autonomic Nervous System Modulation, Cardiac Autonomic Nervous System Research Center of Wuhan University, Wuhan University, Wuhan 430072, China; Hunan Key Laboratory of Two-Dimensional Materials, State Key Laboratory of Chemo and Biosensing, College of Chemistry and Chemical Engineering, Hunan University, Changsha 410082, China; State Key Laboratory of Molecular Engineering of Polymers, Laboratory of Molecular Materials and Devices, Department of Macromolecular Science, Fudan University, Shanghai 200433, China; Key Laboratory of Zhejiang Province for Aptamers and Theranostics, Zhejiang Cancer Hospital, Hangzhou Institute of Medicine (HIM), Chinese Academy of Sciences, Hangzhou 310022, China; College of Chemistry and Molecular Sciences, Key Laboratory of Biomedical Polymers of Ministry of Education, Department of Cardiology, Institute of Molecular Medicine, Renmin Hospital of Wuhan University, State Key Laboratory of Metabolism and Regulation in Complex Organisms, Taikang Center for Life and Medical Sciences, Hubei Key Laboratory of Autonomic Nervous System Modulation, Cardiac Autonomic Nervous System Research Center of Wuhan University, Wuhan University, Wuhan 430072, China; College of Chemistry and Molecular Sciences, Key Laboratory of Biomedical Polymers of Ministry of Education, Department of Cardiology, Institute of Molecular Medicine, Renmin Hospital of Wuhan University, State Key Laboratory of Metabolism and Regulation in Complex Organisms, Taikang Center for Life and Medical Sciences, Hubei Key Laboratory of Autonomic Nervous System Modulation, Cardiac Autonomic Nervous System Research Center of Wuhan University, Wuhan University, Wuhan 430072, China; College of Chemistry and Molecular Sciences, Key Laboratory of Biomedical Polymers of Ministry of Education, Department of Cardiology, Institute of Molecular Medicine, Renmin Hospital of Wuhan University, State Key Laboratory of Metabolism and Regulation in Complex Organisms, Taikang Center for Life and Medical Sciences, Hubei Key Laboratory of Autonomic Nervous System Modulation, Cardiac Autonomic Nervous System Research Center of Wuhan University, Wuhan University, Wuhan 430072, China

**Keywords:** field-effect transistor, point-of-care testing, interfacial engineering, biosensing mechanism, cardiovascular diseases

## Abstract

Point-of-care (POC) testing for electrical detection of cardiovascular disease (CVD) can enable effective screening and surveillance. However, the complexity and diversity of serum samples interfere with the transduction of electrical signals, limiting the sensitivity and accuracy of the biochemical assay. Here, we present the development and performance of an electrical POC assay based on self-assembled lipid-capped transistor sensory interfaces for the simultaneous detection and profiling of five myocardial injury biomarkers, with detection limits as low as pg/mL levels. By reducing Debye screening and non-specific adsorption through phospholipid self-assembly, the electrical assay maintains high sensitivity and specificity in human serum, as well as promising quantitative accuracy. For acute myocardial infarction (AMI) animal modeling, our assay detects variations in key biomarker characteristics within 15 to 45 min after the onset of myocardial injury, with a detection time window more than 30 min earlier than biochemical assays. Through the combination profiling of five biomarker signatures from 265 clinical serum samples, our assay achieves 91.7% accuracy for AMI identification and 79.6% accuracy for cardiovascular disease classification, effectively monitoring the prognosis of AMI patients. This assay may provide reliable clinical guidance for the early diagnosis, risk assessment, and prognosis monitoring of CVD.

## Teaser

HEArT point-of-care assay enables sensitive, rapid cardiovascular identification and clinical management via lipid-capped field-effect transistor biosensors.

## INTRODUCTION

With the prominent issue of uneven distribution of medical resources and the increasing demand for rapid diagnosis, traditional diagnostic technologies that rely on large-scale laboratory equipment and lengthy durations struggle to meet the urgent needs of clinical decision-making and primary medical care [1–4]. As a key innovative achievement in modern medicine, point-of-care (POC) testing technology is reshaping the diagnosis and treatment models for major diseases [5]. Featuring compact and portable advantages, POC devices enable tests to be conducted at the bedside, in community clinics, or even at home, thereby facilitating immediate and personalized diagnosis [6]. Meanwhile, POC technology can efficiently generate reports within minutes to half an hour, allowing doctors to promptly adjust their diagnosis and treatment strategies, significantly reducing the risk of delays in disease management [7,8]. The high-lethality characteristics of cardiovascular disease (CVD), particularly acute myocardial infarction (AMI), impose stringent demands on early-stage diagnostic technologies [9–13]. The prevalence of unhealthy lifestyles has further worsened the suddenness and incidence of CVDs, challenging the time-sensitivity of traditional diagnostic methods [14–16]. With its low cost, portability, and rapid bedside testing capabilities, POC technology is emerging as the primary focus for

innovation in the diagnosis and treatment of CVDs [17]. Monitoring the dynamics of myocardial injury biomarkers (MIBs) offers the potential for ultra-early-stage intervention and individualized treatment of AMI, which is expected to significantly mitigate myocardial injury and the risk of complications, thereby enhancing patient survival rates [15].

With the advantages of label-free rapid detection and easy integration, electrical biosensors can swiftly convert the charge characteristics of biomolecules into measurable electrical signals, which demonstrates great application potential in the field of POC technology [18–25]. Mechanistically, after binding with a fixed recognition element, the biomolecules change the parameters of the electrical sensory interface under an electric field, including conductance/resistance, capacitance, carrier mobility, etc., which in turn transduce into a readable electrical signal [26–31]. However, when applied to complex physiological samples like serum, urine, and interstitial fluid, the POC detection performance of electrical biosensors is significantly limited [32–35]. The narrow electrical double layer (EDL), caused by high ion concentration, restricts the effective interaction between biomolecules and the interface, reducing detection sensitivity [36–38]. Moreover, numerous non-target molecules in physiological media create substantial false signals through electrostatic adsorption, further degrading detection accuracy [39–42]. These challenges severely hinder the widespread application of electrical sensing methods in POC diagnosis of CVDs. Additionally, a ‘black box’ exists in the relationship between biomarker levels and diseases. The overlapping clinical phenotypes of different CVDs and the insufficient specificity of biomarkers urgently necessitate the development of interpretable and accurate data models for rapid screening and risk assessment of CVDs [43–45].

**Figure 1.**
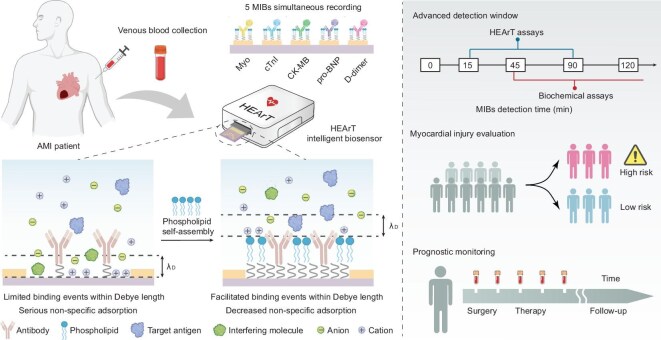
Schematic of the architectural mechanism and clinical application of HEArT POC assay. A HEArT intelligent biosensor is developed for multi-dimensional healthcare of CVD patients *via* venous blood profiling. A self-assembled lipid-capped sensory interface is designed to overcome the limitations of Debye screening and non-specific adsorption in the physiological environment. Composed of multiple FET biosensors based on phospholipid self-assembly, HEArT simultaneously records 5 MIBs in clinical serum samples, including Myo, cTnI, CK-MB, pro-BNP, and D-dimer. Through the combination profiling of detected protein characteristics, HEArT demonstrates clinical applicability in advanced detection time windows, myocardial injury evaluation, and prognostic monitoring of AMI.

To overcome the above limitations, we report a highly sensitive and precise POC assay for MIBs profiling in plasma, termed the High-performance Electrical Assay for CVD Testing (HEArT) (Fig. 1). HEArT consists of a portable detection device, a disposable biosensing array, and an application program. Inspired by natural biological membranes, the HEArT biosensing array comprises ten ‘monolayer phospholipid interface-capped’ field-effect transistors (MPI FETs), enabling simultaneous quantitative analysis of five MIBs, including cardiac troponin I (cTnI), myoglobin (Myo), creatine kinase isoenzyme (CK-MB), N-terminal pro-brain natriuretic peptide (pro-BNP), and D-dimer. Self-assembled phospholipid molecules are densely arranged on the electrical sensory interface to create an ion-conductive barrier layer, effectively expanding the range of the EDL. This configuration allows more binding events within the EDL, enhancing detection sensitivity under high ionic strength. Additionally, the zwitterionic groups at the hydrophilic terminals form a hydration layer above the phospholipids, reducing nonspecific adsorption and ensuring anti-fouling capability in physiological environments. This ‘bifunctional interface capping’ strategy provides an effective approach to enhance detection performance in body fluids, promising to overcome the limitations of traditional electrical assays in POC technology. By introducing a linear discriminant analysis (LDA) algorithm, multi-biomarker features are integrated to construct a myocardial injury signature (MI_sig_), establishing an interpretable and accurate data model for risk evaluation and rapid screening of CVDs.

The HEArT POC assay we developed achieves precise detection of pg/mL level MIBs in clinical serum samples, with quantitative accuracy comparable to enzyme-linked immunosorbent assay (ELISA). Benefiting from its excellent detection capability, HEArT can identify significant changes in MIBs within 15 min after establishing an AMI model in beagles, more than 30 min earlier than biochemical assays. Through analysis and validation of 265 clinical serum samples, HEArT achieved a sensitivity of 76.5% and specificity of 97.0% for identifying AMI, with a classification accuracy of 79.6% for different CVDs, demonstrating its capability to assess risks in clinical chest pain patients. Furthermore, we utilized HEArT to continuously record and analyze MIB levels in AMI patients undergoing percutaneous coronary intervention (PCI) over a 7-d period, successfully enabling prognosis monitoring and early warning of myocardial ischemia-reperfusion injury (MIRI). In addition, we developed a portable HEArT POC system with simple operation and low cost. The HEArT system can output detection and diagnostic results on a mobile app within 15 min of obtaining a serum sample, showing excellent clinical applicability. These results are of great significance for highly sensitive and precise profiling of biomarkers in physiological environments, holding promise for providing reliable clinical guidance for early diagnosis, risk assessment, and prognosis monitoring of CVD.

## RESULTS

### Design, characterization, and mechanism of MPI FET biosensors

**Figure 2.**
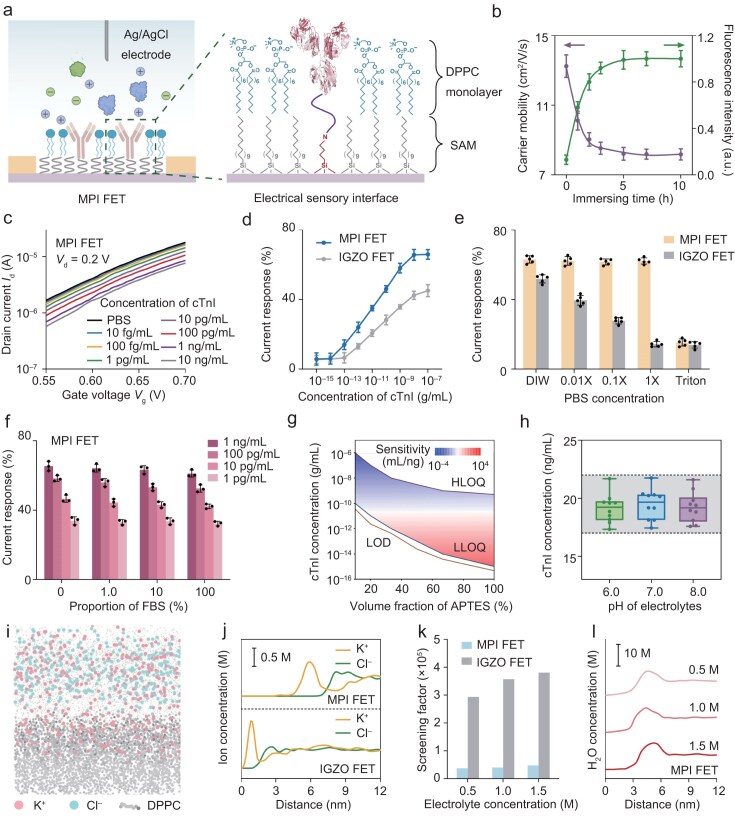
Characterization and molecular mechanisms of MPI FET biosensors. (a) Molecular architectures of the liquid-gated MPI FET and the electrical sensory interface. DPPC monolayer and cTnI antibodies were modified at the IGZO interface with the assistance of SAM. (b) Carrier mobility and fluorescence intensity of MPI FET under different immersing times in 0.5 mg/mL DPPC suspension. The alkyl chains of phospholipids were labeled with Dil. (c) *I*_d_-*V*_g_ curves of MPI FETs in response to different concentrations of cTnI. The source-drain voltage was set at 0.2 V. (d) Comparison of current responses of MPI FET and IGZO FET toward different concentrations of cTnI. The gate voltage was set at 0.6 V. (e) Current responses of MPI FET and IGZO FET toward 1 ng/mL cTnI in DIW, different concentrations of PBS, and Triton. (f) Current responses of MPI FET toward cTnI under different proportions of FBS. (g) Diagram of analytical quality factors determined by the ratio of APTES to OTES, including LOD, LLOQ, HLOQ, and sensitivity. (h) Recovery of MPI FET toward 20 ng/mL cTnI under different pH levels of electrolytes. The box plots show the median, interquartile range, and extreme values of 10 technical replicates. The recovery rate between 85% and 110% is labeled in gray. (i) Schematic of the molecular dynamics model. A stable EDL is formed on the self-assembled lipid-capped sensory interface. (j) Ion distribution (K^+^ and Cl^−^) above the surface of MPI FET and IGZO FET in 0.5 M KCl electrolyte. An ion-free layer is attached to MPI FET with a height of ∼4 nm. (k) Comparison of screening factors at 7 nm above the interface of MPI FET and IGZO FET. The electrolyte concentrations were 0.5 M, 1.0 M, and 1.5 M, respectively. (l) H_2_O distribution above the surface of MPI FET under different electrolyte concentrations. H_2_O molecules gather at ∼4 nm above the interface. Data in (b), (d), (e), and (f) are expressed as mean ± s.d. for 5 technical replicates.

Figure 2a shows the molecular architectures of the MPI FET biosensor targeting cTnI and its sensory interface. A self-assembled monolayer (SAM) composed of aminopropyl triethoxysilane (APTES) and octyltriethoxysilane (OTES) was modified on the 25 nm-thick indium gallium zinc oxide (IGZO) channel surface to immobilize cTnI antibodies with the assistance of glutaraldehyde linkers. Subsequently, cTnI antibody-modified IGZO-FET was immersed in a dipalmitoyl phosphatidylcholine (DPPC) vesicle suspension, and the phospholipid molecules were assembled on the surface of the channel material (Fig. S1). Figure S2 presents the transfer characteristic curves of MPI FETs under different immersing times. It can be observed that the carrier mobility of MPI FETs gradually decreased with the assembly of the phospholipid layer, which can be attributed to the increasing capacitance of the sensory interface (Fig. 2b) [46,47]. The carrier mobility reaches a constant value after being immersed for 5 h, indicating that phospholipid molecules have completely covered the surface of the IGZO channel. We then labeled the alkyl chains with Dil and cross-verified the phospholipid layer formation and coverage *via* confocal fluorescence microscopy (Fig. S3). The fluorescent dye was distributed uniformly on the surface of the IGZO, and the fluorescence intensity reached the maximum after being immersed for 5 h, which was consistent with the electrical measurements. The presence of absorption peaks of the phosphate skeleton (1238 cm^–1^) and amide bond (1536 cm^–1^ and 1648 cm^–1^) in attenuated total reflection Fourier transforms infrared spectroscopy (ATR-FTIR) and the characteristic peaks of C 1s (286.7 eV), N 1s (399.8 eV), S 2p (162.2 eV), and P 2p (132.3 eV) in X-ray photoelectron spectroscopy (XPS) suggest the successful modification of DPPC and cTnI antibodies (Figs S4 and S5) [48,49]. Atomic force microscopy (AFM) revealed the uniform assembly of the phospholipid functionalized layer on the IGZO surface with an average height of ∼4.0 nm (Fig. S6). This average height is consistent with the theoretical molecular length of phospholipids, indicating that DPPC is self-assembled in the form of a monolayer. The high uniformity of the phospholipids is mainly attributed to the introduction of the hydrophobic SAM layer [50–52]. As shown in Fig. S7, the contact angles of the IGZO surface are 83.8° and 113.2° before and after SAM functionalization, respectively. The alkyl chain of OTES can interact with the hydrophobic tail to induce the directional assembly of DPPC. The average height of cTnI antibodies on the surface of the MPI FETs was ∼2–3 nm, which is closer to the sensing surface compared with IGZO FETs (∼6–8 nm) (Fig. S8). The reason lies in the fact that the densely assembled phospholipid layer narrows the distance between antibody molecules and the sensory interface.

To investigate the detection performance of the biosensors before and after phospholipid assembly, we measured the *I*_d_−*V*_g_ transfer characteristic curves of IGZO FET and MPI FET in different concentrations of cTnI under optimized antibody modification concentration and incubation time (Fig. S9). As shown in Fig. 2c and Fig. S10, with the increase of cTnI concentration, the *I*_d_ of the biosensor decreases constantly. The variations in current can be attributed to the binding of the sensory interface with cTnI antigens. Specifically, the surface negative charge accumulates in the n-type IGZO channel as the negatively charged cTnI antigen binds to the sensory interface, which in turn leads to a reduction in carrier density and conductivity, thereby reducing the current under the same *V*_g_. The current response (100% × Δ*I*/*I*_o_), which is defined as the ratio between the variation in *I*_d_ (Δ*I*) and the initial current (*I*_o_), is used to evaluate the response signal of the FET biosensors. As shown in Fig. 2d, MPI FETs exhibited significantly higher current responses to cTnI and presented a wider detection range and lower detection limits compared with IGZO FETs. This phenomenon can be ascribed to more binding events being incorporated into the Debye layer after the modification of phospholipids, which induces more charge variation at the sensory interface. In addition, unlike IGZO FETs, the current responses of MPI FET did not increase at high cTnI concentrations, which indicates the inhibition of non-specific adsorption to a certain extent. Figure 2e presents the current responses of MPI FET and IGZO FET toward 1 ng/mL cTnI in deionized water (DIW) and phosphate buffer saline (PBS) at different concentrations. Unlike IGZO FET, whose sensing capability is highly dependent on ionic strength, the current responses of MPI FET are within the margin of error. When the integrity of the phospholipid monolayer was destroyed by Triton, MPI FETs exhibited similar sensing capabilities as IGZO FETs. It can be observed that with the increase of DPPC coverage, the signal attenuation of the sensing interface under high ionic strength gradually decreases (Fig. S11). These outcomes indicated that the dense assembly of phospholipids is key to maintaining the sensing performance of MPI FETs under high ionic strength.

Subsequently, we explored the detection performance of MPI FETs in the physiological environment. Both biosensors were exposed to different proportions (0%, 1.0%, 10%, 100%) of fetal bovine serum (FBS), and the current responses toward cTnI were measured (Fig. 2f and Fig. S12). Although signal attenuations were observed in both biosensors, the current responses of the MPI FET seem constant. Even in 100% of the FBS, the signal attenuation of MPI FETs for 1 ng/mL cTnI was only 6.5%, compared to ∼63.1% for IGZO FETs. This is because the zwitterionic groups at the hydrophilic end of phospholipids can induce the formation of a dense hydration layer on the electrical sensory interface, effectively inhibiting the non-specific adsorption of charged biomolecules, thus ensuring signal stability of the FET biosensors in the physiological environment (Fig. S13). Encouraged by the robustness of MPI FET, we measured and plotted the cTnI’s response calibration curves in FBS (Fig. S14). We calculated analytical quality factors, including the limit of detection (LOD), sensitivity, and detection range defined by the lowest limit of quantitation (LLOQ) and the highest limit of quantitation (HLOQ) to assess the impact of the ratio of APTES to OTES on detection capability (Fig. 2g). Considering that the clinical standard value of cTnI is ∼20 ng/mL, we selected the MPI FETs with the highest sensitivity around the standard value (33 vol% of APTES) to evaluate cTnI levels. Next, we measured the current responses of 30 MPI FETs toward 20 ng/mL of cTnI in FBS and calculated the corresponding reported concentrations. As graphically presented in Fig. 2h and Fig. S15, the recovery rate of MPI FETs ranged from 85% to 110% at the ng/mL level and was immune to pH fluctuations within a certain range. In addition, the current responses of MPI FETs could be maintained at 4°C for 7 d (Fig. S16). These results indicate that MPI FET can achieve high stability and precise quantification of cTnI in serum samples.

In order to reveal the mechanism of the improved detection performance of MPI FETs, we investigated the ion distribution at the sensory interface of MPI FET and IGZO FET by molecular dynamics (MD) simulations (Fig. 2i). We first explored the distribution of N atoms on the MPI FET interface (Fig. S17). N atoms exist only in the tail of DPPC, which can reflect the occupied length of the phospholipid at the electrical sensory interface. The distribution of N atoms in different electrolytes is basically the same, indicating that phospholipids can maintain structural stability at high ionic strength. Furthermore, we computed the distribution of K^+^ and Cl^−^ in different electrolytes at the sensory interface of MPI FET and IGZO FET, as graphically presented in Figs S18−S20. A stable Helmholtz layer and a diffusion layer were formed above the interface of both biosensors, which is consistent with the Gouy–Chapman EDL model. In comparison, the ions above the MPI FET are significantly further away, since an ion-free layer is closely attached to the sensory interface with a height comparable to the length of phospholipid molecules (Fig. 2j and Fig. S21). To quantify the degree of charge screening, the relationship between the calculated screening factor and the distance to the sensory interface is plotted in Fig. S22. Compared with IGZO FETs, the charge screening of MPI FETs is initiated at a further distance from the sensory interface, indicating that the presence of phospholipids effectively mitigates the Debye screening effect. Meanwhile, the potholed surface of MPI FET results in a looser aggregation of ions, ultimately compromising the charge screening of the analytes. The reduction of charge screening and the extension of the Debye length promote more binding events of target and recognition elements within the Debye layer. Figure 2k plots the screening factors at 7 nm (measured average height of cTnI antibodies) from the sensing interface. The screening factor of IGZO FET increased rapidly with ionic strength, while MPI FETs altered little, which explains the stable current responses of MPI FET under different ionic strengths. This change in the interface screening effect is also reflected in the zeta potential of the biosensing surface (Fig. S23). We also calculated the variation of the screening factor at different DPPC coverage degrees in the MD simulation (Fig. S24). It can be observed that the screening factor increases rapidly as the coverage degree decreases. This might be due to the ions in the solution system that aggregate on the uncovered electrode surface under the action of the electric field. When the coverage degree drops below 40%, the screening factor tends to stabilize, indicating that the effect of phospholipids can be ignored at this point. The interface after phospholipid functionalization showed the greatest change in zeta potential after antigen binding, indicating that more charges entered the Debye length range. In addition, due to the presence of zwitterionic groups, a large amount of water molecules gather around the DPPC (Fig. 2l). The formation of the hydration layer reduces the surface binding energy of non-specific biological molecules. The results of ultraviolet-visible spectroscopy analysis indicate that the interface functionalized with phospholipids exhibits better anti-fouling capabilities for both positively (lysozyme) and negatively (serum albumin) charged biological molecules (Fig. S25). Quartz Crystal Microbalance (QCM) was used to quantitatively detect the adsorption amount of non-target protein (bovine serum albumin) at the interfaces of MPI FET and IGZO FET (Fig. S26). The results show that the adsorption amount of fibrinogen at the MPI FET interface is 7.8 ng/cm², which is much lower than 85.5 ng/cm² at the IGZO FET interface, proving that the phospholipid hydration layer can effectively inhibit non-specific protein adsorption. This can effectively improve the anti-fouling capability of the sensory interface, reducing non-specific adsorption and ensuring quantitative reproducibility and precision in the physiological environment.

### Advanced detection time window *via* HEArT

**Figure 3.**
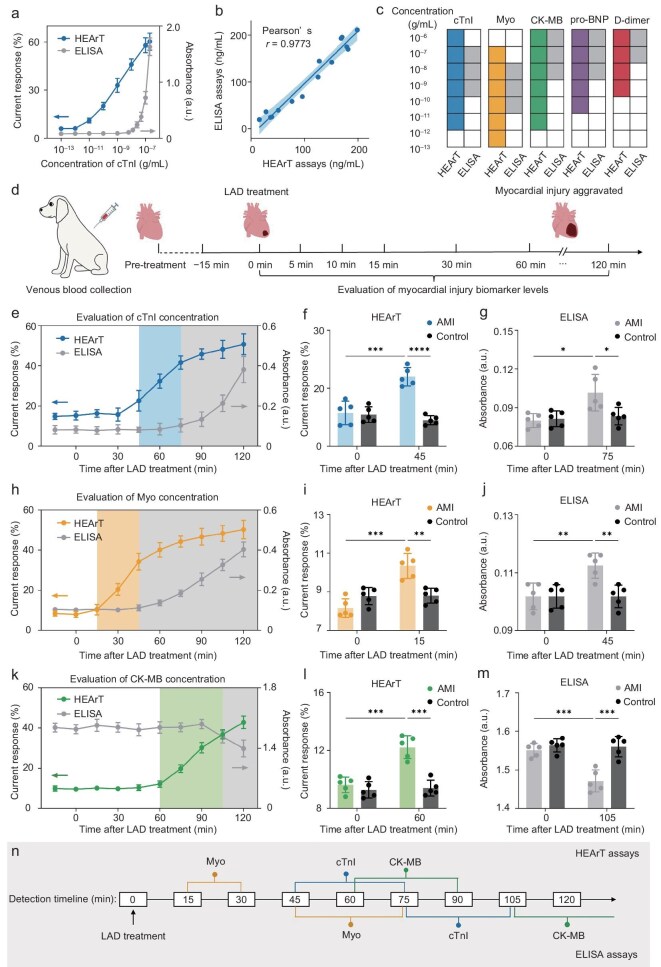
Evaluation of AMI detection time window *via* HEArT assay. (a) Comparison of detection capabilities of HEArT and ELISA assays targeting different concentrations of cTnI. (b) Correlation of the measurement results of HEArT and ELISA assays targeting cTnI (Pearson correlation coefficient, *r* = 0.9773). (c) Effective quantitative ranges of HEArT and ELISA assays targeting 5 MIBs. HEArT exhibits wider dynamic ranges compared with the ELISA assay. (d) Schematic illustration of detection time window evaluation of AMI modeling beagles, including LAD treatment, venous blood collection, and recording MIB levels. (e, h, k) Evaluation of (e) cTnI, (h) Myo, and (k) CK-MB in serum of an AMI modeling beagle *via* HEArT and ELISA assays. The detection time window of HEArT is labeled in color, while the detection time window of ELISA is labeled in gray. (f, i, l) Comparison of current responses of (f) cTnI, (i) Myo, and (l) CK-MB in AMI modeling and control beagle serum samples by HEArT assay. The variations in current responses of cTnI, Myo, and CK-MB can be observed at 45, 15, and 60 min after LAD treatment, respectively. (g, j, m) Comparison of absorbance of (g) cTnI, (j) Myo, and (m) CK-MB in AMI modeling and control beagle serum samples by ELISA assay. The variations in absorbance of cTnI, Myo, and CK-MB can be observed at 75, 45, and 105 min after LAD treatment, respectively. (n) Detection time window of cTnI, Myo, and CK-MB in venous serum of AMI modeling beagles comparing HEArT with ELISA assays. Data in (a) are expressed as mean ± s.d. for 3 technical replicates. Data in (e‒m) are expressed as mean ± s.d. for 5 technical replicates. Statistical differences were determined by unpaired two-sided *t*-tests. *P < 0.05; **P < 0.01; ***P < 0.001; ****P < 0.0001.

Motivated by the excellent detection capability in serum samples, we further explored the applicability of HEArT in the diagnosis of AMI. The diagnosis of AMI usually relies on the comprehensive analysis of multiple MIB levels [15,53–55]. Therefore, a multi-channel HEArT biosensing array was built for the simultaneous recording of 5 MIBs (Fig. S27). In addition to the above-mentioned MPI FET for cTnI detection, we also constructed MPI FETs targeting Myo, CK-MB, pro-BNP, and D-dimer. The detection range of each MIB was optimized according to the strategy of adjusting the ratio of OTES in Fig. 2g, with no crosstalk being observed among the multiple channels (Fig. S28). The LOD, HLOQ, and LLOQ for each MIB are summarized in Table S1. We then compared the detection capabilities of HEArT with ELISA, which is clinically regarded as the gold standard for quantitative biochemical assays for MIBs. As indicated in Fig. 3a, the LOD of HEArT assay for cTnI detection was 4.2 × 10^−12^ g/mL, which was >100 times lower than that of ELISA (6.3 × 10^−9^ g/mL). In addition, HEArT also exhibited a wider dynamic range (∼5 orders of magnitude) than the ELISA assay (<3 orders of magnitude). Further, we prepared 15 simulated serum samples within the detection range of ELISA by the standard addition method, and quantified cTnI concentrations by both HEArT and ELISA assays, respectively (Fig. 3b). According to Pearson correlation analysis, the detection results of the two methods exhibited a good linear correlation (*r* = 0.9773), indicating that HEArT had a comparable quantitative capability as ELISA assays. Similar results were also obtained for the detection of Myo, CK-MB, BNP, and D-dimer (Figs S29 and S30). The effective quantitative ranges of both assays for different MIB detection are listed in Fig. 3c. Interference studies confirm no significant signal deviation (<10%) from hemolysis (hemoglobin, 1 μg/mL), icterus (bilirubin, 1 μg/mL), lipemia (triglycerides, 1 μg/mL), or common drugs (aspirin, clopidogrel, and β-blockers, 10 μg/mL) (Fig. S31). These results indicate that HEArT shows higher sensitivity and a wider detection range for MIB profiling, and exhibits a quantitative capability comparable to ELISA.

As we mentioned above, early screening is one of the most urgent challenges in AMI diagnosis. Therefore, we further explored the detection time window of HEArT for AMI diagnosis. We established beagle AMI models by ligation of the left anterior descending coronary artery (LAD), while a sham operation beagle was used as the negative control. The structure of the heart and the patterns of biomarker changes in beagles are highly similar to those in humans. Venous serum samples were collected at intervals of 30 min before and 120 min after LAD treatment, and the concentration of 5 MIBs was recorded by both HEArT and ELISA assays (Fig. 3d). Figure 3e exhibits the concentration variations of cTnI in beagle serum before and after LAD treatment. The concentration of cTnI in the serum of the LAD-treated beagle increased gradually within 120 min, which was different from the control group, with no significant changes seen after the sham operation (Fig. S32). Quantitative analysis showed that the current response of HEArT at 45 min after AMI model establishment was significantly higher than that before LAD treatment (*P* = 0.0007) and the control group (*P* < 0.0001) (Fig. 3f). Meanwhile, a significant change in absorbance was observed at 75 min after LAD treatment for ELISA assays (Fig. 3g). Similar results were obtained when evaluating cTnI levels in the venous serum of other LAD-treated beagles (Fig. S33). The statistical results showed that the increase in cTnI levels could be detected by HEArT within 45−75 min after the establishment of the AMI model, while for ELISA was 75−105 min. We then evaluated the serum Myo and CK-MB levels using the same strategy (Fig. 3h and k). HEArT could report significant increases in Myo and CK-MB at 15 and 60 min after LAD treatment (Fig. 3i and l), respectively, compared with 45 and 105 min by ELISA assays (Fig. 3j and m). Through statistical analysis of 4 LAD-treated beagles, the detection time window of HEArT was more than 30 min earlier than that of ELISA assays (Fig. 3n, Figs S34 and S35). This advanced detection time window is mainly attributed to the lower detection limit of HEArT. In the early stage of AMI, the degree of myocardial damage is mild, and the concentration of MIBs in the blood is lower than the LOD of ELISA. Therefore, the variations in MIB concentration would be ignored by the biochemical assays. With the aggravation of myocardial injury, more MIBs are released into the blood, at which point changes in the absorbance signal can be observed. The significantly improved sensitivity of HEArT could effectively report the variations in low-concentration MIBs, resulting in a shorter diagnostic window compared to ELISA. For pro-BNP and D-dimer quantification, no persistent variations were observed by both HEArT and ELISA assays within 120 min, indicating that these two MIBs contributed little to the early screening of AMI (Figs S36 and S37). The above results indicate that HEArT could rapidly and precisely report the changes of trace MIBs, advancing the detection time window of AMI, and exhibiting promising application potential in the field of early AMI screening.

### Myocardial injury evaluation of CVD patients *via* HEArT

**Figure 4.**
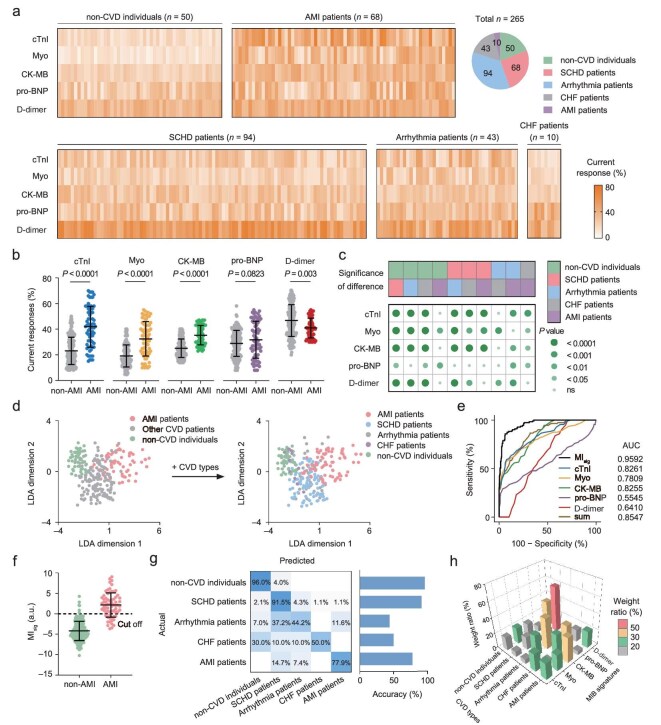
Risk evaluation and rapid screening of CVD patients *via* HEArT assay. (a) Heat maps of five MIB signatures in clinical serum samples obtained by HEArT. (*n* = 265; that is, 50 for non-CVD individuals, 68 for AMI patients, 94 for SCHD patients, 43 for arrhythmia patients, and 10 for CHF patients). The color intensity indicates the expression level of MIB. (b) MIB expression levels in clinical serum samples from AMI patients (*n =* 68) and non-AMI individuals (*n* = 197). (c) Differences in MIB signatures for distinguishing patients with two different types of clinical diagnosis. (d) Two-step LDA algorithm for multiclass assessment of CVD patients. The first step discriminated between AMI patients and non-AMI individuals, while the second step classified different CVD types. Both steps use 5 MIB signatures as input, and the result of each step was visualized on a two-dimensional plane. (e) ROC curves and corresponding AUC when using different signatures as criteria to differentiate AMI patients from non-AMI individuals. MI_sig_ demonstrates the highest accuracy in AMI diagnosis with its largest AUC value. (f) AMI identification results *via* MI_sig_ obtained by the LDA algorithm. The cut-off value of MI_sig_ was set as 0. (g) Confusion matrix summarizing the CVD classification results. The prediction accuracy of each CVD type is presented in a histogram. (h) The weight ratio of five MIB signatures in LDA algorithms for identifying specific CVD types. Data in (b) and (f) are expressed as mean ± s.d. for the indicated number of biological replicates. Statistical differences were determined by paired two-sided *t*-tests. ns, not significant.

Considering that the clinical symptoms of AMI are similar to those of other CVDs, an accurate and effective diagnosis of CVD is essential for patient risk assessment and specific treatment. Next, we explored the clinical applicability of HEArT in AMI identification and CVD diagnosis. The levels of MIB expression in serum from 265 clinical chest pain patients (including 50 non-CVD individuals, 68 AMI patients, 94 patients with stable coronary heart disease (SCHD), 43 patients with arrhythmia, and 10 patients with chronic heart failure (CHF)) were measured by HEArT assay and summarized in Fig. 4a. Although it can be observed that MIB expression levels are generally higher in AMI patients than in non-AMI individuals, there is an overlap in current response between AMI and non-AMI individuals, which makes it challenging to accurately discriminate between AMI and non-AMI individuals based on a single biomarker criterion (Fig. 4b). Additionally, the sum of current responses cannot accurately identify AMI patients either (Fig. S38). Furthermore, significance tests showed that different CVDs were difficult to effectively discriminate due to the lack of significant heterogeneity in MIB expression among CVD patients (Fig. 4c).

To achieve effective AMI identification and CVD classification, a two-step linear discriminant analysis (LDA) algorithm was employed to establish the relationship between MIB characteristics and CVD in the training cohort (Fig. 4d). First, we considered the weighted aggregation of multiple MIBs, determined the optimal weights of each biomarker by the LDA algorithm, and calculated the myocardial injury signatures (MI_sig_). A higher value of MI_sig_ indicates that the patient may suffer from more severe myocardial injury and a higher risk of AMI occurrence. For individual MIBs and their combinations, we constructed receiver operating characteristic (ROC) curves and precision-recall (PRC) curves for AMI identification (Fig. 4e and Fig. S39). The area under the curve (AUC) and average precision of MI_sig_ were significantly higher than those of individual MIBs and SUM, indicating that MI_sig_ was more effective in identifying AMI. In each ROC curve, we identified a cut-off value to maximize the sum of sensitivity and specificity. As shown in Fig. 4f, MI_sig_ can identify AMI with an accuracy of 91.7% (95% confidence interval (CI): 88.4%–95.0%), with sensitivity and specificity of 76.5% (95% CI: 71.4%–81.6%) and 97.0% (95% CI: 94.9%–99.1%), respectively, much higher than those of using a single MIB criterion (Fig. S40). We then asked if HEArT could effectively classify different types of CVD. We classified other CVD patients according to clinical diagnosis results and constructed a second-step LDA algorithm using the CVD types as input (Fig. 4d). The classification results were mapped onto a two-dimensional plane and then quantitatively summarized in a confusion matrix (Fig. 4g). HEArT achieved the identification of non-CVD individuals, SCHD patients, arrhythmia patients, CHF patients, and AMI patients with an accuracy of 96.0%, 91.5%, 44.2%, 50.0%, and 77.9%, respectively, and an overall accuracy of 79.6% (95% CI: 74.7%–84.5%) was obtained for multiclass CVD diagnosis. Although single markers could effectively discriminate one or two types of CVD, the accuracy of AMI identification and CVD classification achieved by combining 5 MIBs is significantly higher than that of any single biomarker (Fig. S41).

Moreover, we investigated the relationship between the MIB signatures and CVDs. For each type of CVD, we constructed LDA models for specific disease discrimination. Figure 4h shows the absolute value of the weight ratio for MIB signatures in each disease identification. The higher the weight ratio, the greater the MIB’s contribution to identifying the corresponding CVD. It can be observed that the weight ratios of cTnI, Myo, CK-MB, and D-dimer in AMI identification are 21.5%, 20.8%, 33.2%, and 20.2%, respectively, suggesting that the above 4 MIBs contribute to the identification of AMI. Meanwhile, the weight ratio of the D-dimer signature reached 61.2% in SCHD identification, indicating the high specificity of the biomarker for SCHD. We further verified the weight stability through 5-fold cross-validation in the training set. The results show that after 20 cross-validations, the coefficient of variation of each marker’s weight is <10%, indicating the robustness of the weight analysis (Table S2). The overall accuracy of both AMI identification and CVD classification will be reduced if any MIB signature is excluded from the LDA algorithm, suggesting the rationality of the combination consideration of the 5 biomarkers (Fig. S42). The above results indicated that, combined with data modeling analysis, HEArT could be used to establish the relationship between MIB signatures and CVDs, leading to the accurate identification of CVDs.

### Prognosis monitoring *via* HEArT

The poor prognosis and high recurrence of AMI severely limit the postoperative cure rate and survival rate of patients. In particular, MIRI is a common malignant prognosis in AMI patients after interventional therapy. Specifically, when the coronary artery occlusion is cleared, the blood supply to ischemic cardiomyocytes is restored, and the myocardium instead exhibits progressive and aggravated damage. MIRI may lead to myocardial edema and bleeding, severe malignant arrhythmias, and even sudden cardiac death, with a mortality rate of >10% [56–58]. Continuous monitoring of protein signatures in patients with AMI can provide critical information about prognostic progression. Particularly, recording variations in MIB levels provides the possibility for early warning and timely treatment of MIRI.

**Figure 5.**
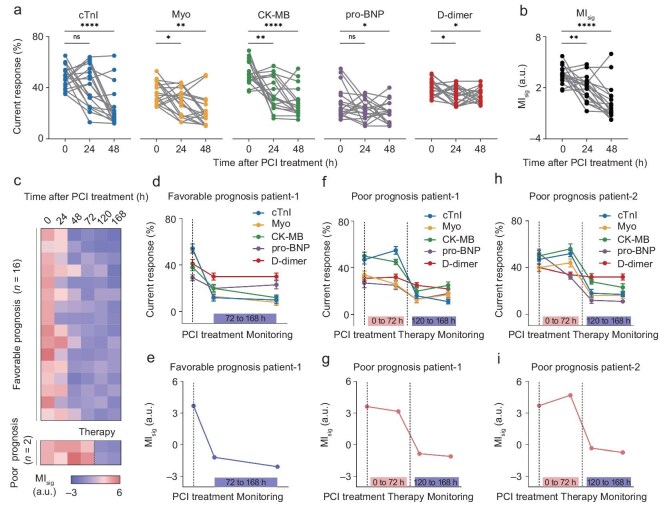
Prognosis monitoring of AMI patients *via* HEArT assay. (a) Variations of MIB signatures in serum samples of AMI patients 24 h and 48 h after PCI treatment (*n =* 18). Significant changes were observed 48 h after surgery in each MIB level. (b) Variations of MI_sig_ of AMI patients 24 h and 48 h after PCI treatment (*n =* 18). The overall level of MI_sig_ decreased significantly 48 h after surgery. (c) Longitudinal recording of MI_sig_ of AMI patients within 168 h after PCI treatment (*n =* 18; that is, 16 for favorable prognosis patients and 2 for poor prognosis patients). The levels of MI_sig_ were summarized by heat maps. (d and e) Variations of (d) serum MIB expression levels and (e) corresponding MI_sig_ in a favorable prognosis patient within 168 h after PCI treatment. The MI_sig_ returned to the normal level within 72 h, and no upward trend was seen within 168 h. (f–i) Variations of (f, h) serum MIB expression levels and (g, i) corresponding MI_sig_ in two poor prognosis patients within 168 h after PCI treatment. The MI_sig_ maintained high levels at 72 h and decreased after intervention therapy. Data in (d), (f), and (h) are expressed as mean ± s.d. for 3 technical replicates. Statistical differences were determined by paired two-sided *t*-tests. ns, not significant; *P < 0.05; **P < 0.01; ***P < 0.001; ****P < 0.0001.

To evaluate the effectiveness of PCI in alleviating myocardial injury, we used HEArT to track MIB characteristics before and after PCI treatment in 18 patients with AMI, including measuring venous blood samples collected 12 h before and 48 h after surgery and calculating corresponding MI_sig_ values. Figure 5a shows the variations of MIB levels within 48 h after PCI. For cTnI, 38.9% (7/18) of patients experienced a decrease in marker concentration within 24 h after PCI, while rising to 83.3% (15/18) within 48 h. Although similar statistical results were shown when measuring the other four MIBs, the level of marker expression indicated significant patient specificity. In order to reflect the prognosis situation more intuitively, the corresponding MI_sig_ value was calculated and presented in Fig. 5b. The overall level of MI_sig_ in AMI patients decreased significantly 48 h after surgery, indicating that the degree of myocardial damage was generally alleviated after PCI treatment.

Furthermore, we recorded the MIB characteristics of patients longitudinally within 168 h after PCI treatment (Fig. S43), and the calculated MI_sig_ values are listed in Fig. 5c. It can be observed that the MI_sig_ values of 16 patients returned to normal levels within 72 h, and no upward trends were observed within 168 h, indicating the favorable prognosis of these patients. Figure 5d and e present the changes in MIB levels and MI_sig_ in one patient with a favorable prognosis. However, MI_sig_ values in two patients maintained a high level (greater than the cut-off value) 72 h after surgery, suggesting the possibility of MIRI in these two patients after PCI treatment (Fig. 5f–i). Clinical examination results showed ST-segment changes in the electrocardiogram (ECG) of these two patients, confirming the HEArT’s prediction of MIRI risk (Fig. S44). After timely treatment and intervention (including the use of β-blockers, etc.), the MI_sig_ values of both patients decreased within 48 h, suggesting the effective remission of MIRI. The above results indicate that the continuous monitoring of MIB characteristics by HEArT provides an effective strategy for the prognosis assessment of AMI, which can help in the timely judgment of malignant prognosis reactions and clinical strategies.

### HEArT device for multi-dimensional CVD management

**Figure 6.**
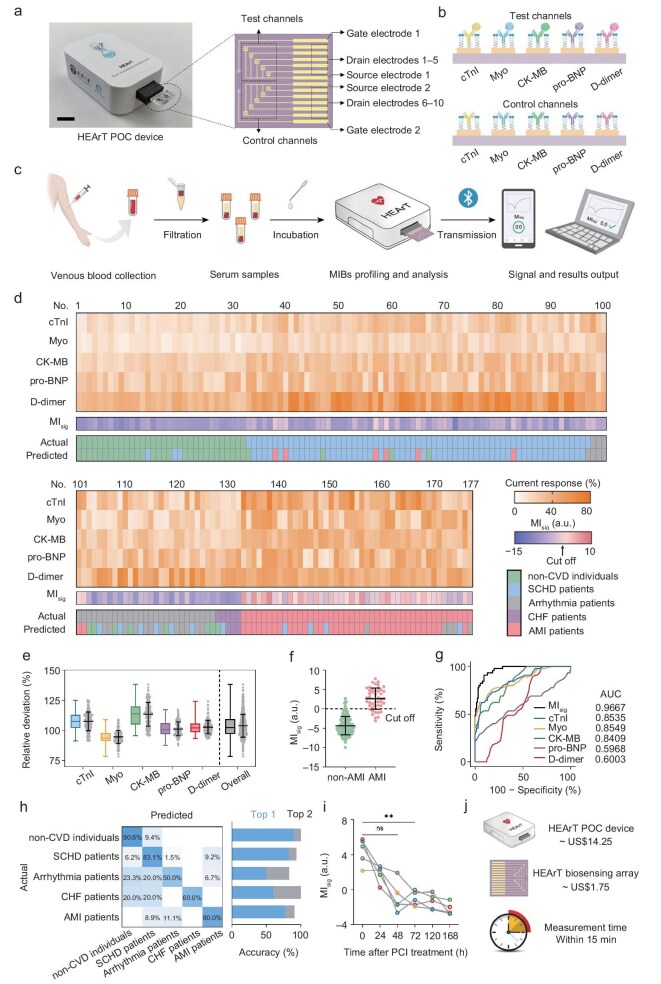
Clinical applicability of the HEArT POC system. (a) Schematic diagram of the HEArT POC system. The HEArT POC system is composed of a POC device, FET biosensing arrays, and intelligent terminal-based application programs (scale bar: 2 cm). (b) Schematic diagram of the test channels and control channels of HEArT biosensing arrays. (c) Workflow of the HEArT assay using the POC device, including sample collection, MIBs detection, data analysis, and result output. (d) Heat maps of MIB signatures, MI_sig_, and predicted CVD types in the validation cohort of 177 clinical serum samples obtained by HEArT. The color intensity indicates the expression levels of MIB. (e) Comparison of quantitative results of 5 MIBs in clinical serum samples using HEArT assay and ELISA assay (*n* = 177). (f) AMI identification results *via* MI_sig_ obtained by the HEArT POC device in the validation cohort (means ± s.d.). The cut-off value of MI_sig_ was set as 0. (g) ROC curves of AMI identification *via* the HEArT POC system. (h) Confusion matrix summarizing the CVD classification results in the validation cohort. The prediction accuracy of each CVD type is presented in a histogram. (i) Continuous monitoring of the MI_sig_ values of 5 AMI patients within 168 h after PCI treatment. (j) Cost and measurement time of the HEArT POC system. HEArT could complete a single test within 15 min at a cost of <$2 per test. Data in (d), (f), and (i) are obtained from the HEArT POC system. Statistical differences were determined by paired two-sided *t*-tests. ns, not significant; **P < 0.01.

To achieve POC diagnosis and remote healthcare, we developed a portable HEArT POC system that rapidly obtains and outputs multi-dimensional information on CVDs through serum MIB levels. The HEArT POC system consists of a reusable POC device, a disposable multi-channel biosensing array, and an intelligent application program featuring analytical algorithms (Fig. 6a). Measuring 10.0 cm (L) × 6.7 cm (W) × 3.5 cm (H), the HEArT POC device integrates a microcontroller unit, a digital-to-analog converter, a transimpedance amplifier, a battery system, and a Bluetooth module, facilitating data acquisition, information processing, and signal transmission (Fig. S45). The 14-to-1 multiplexer works with the 10-channel HEArT array and sequentially accesses the measured current signals from the FET array. The HEArT biosensing array is symmetrically divided into test channels and control channels (Fig. 6b). Each group of channels is modified using five MIB antibodies, respectively, to enable self-calibration in a single measurement. The excellent repeatability and low inter-batch variation of the HEArT array (with a relative standard deviation of 5.16%) further guarantee the accuracy of the measurement results (Fig. S46).

Figure 6c illustrates the user-friendly and easy-to-operate workflow of the HEArT POC system, which comprises two simple steps: (i) slowly add 100 μL of freshly separated human plasma to the test channels of the HEArT array while simultaneously adding iso-osmotic PBS (20 nM) to the reference channels. (ii) Incubate the HEArT array at room temperature (25°C) for 10 min, and then evaluate the transfer characteristic curves of the FET biosensor using a smartphone or personal computer terminal APP. As the analysis algorithm shown in Fig. 4 is pre-integrated into the APP, the HEArT POC system can directly provide the analysis and diagnosis results, including the current response of each MIB, the calculated MI_sig_, and the predicted probabilities of four CVDs. In order to achieve the real-POC testing, we rapidly isolated fresh serum through filtration without the need for laboratory equipment. We optimized the filter membrane material and surface properties, achieving a MIB permeability of >98% and the removal of blood cells (Fig. S47). Notably, the HEArT POC system can be easily operated by untrained personnel without the need for fully equipped laboratories or highly complex analytical techniques, and delivers analysis results within 15 min of sample collection, facilitating rapid and portable POC body-fluid diagnosis (Video S1 and Video S2).

We further verified the detection accuracy and clinical applicability of the HEArT POC system. We first conducted a double-blind test on 177 serum samples from patients with clinically suspected CVDs and recorded the output results of the HEArT POC system, including current responses of single MIBs, the MI_sig_ values, and the predicted CVD type (Fig. 6d). Figure 6e compares the quantitative results of MIBs in serum given by HEArT and ELISA assays. The deviation between the measurement results of both assays ranges from 82.4% to 130.2% (95% CI: 95.4%–108.3%). The measurement deviations for Myo, cTnI, CK-MB, pro-BNP, and D-dimer are 90.5% to 125.4%, 82.4% to 108.9%, 95.6% to 130.2%, 87.5% to 118.4%, and 92.6% to 126.3%, respectively. Similarly, the spiked recovery rates of the five biomarkers in the serum for HEArT ranged from 84.0% to 114.7% (Table S3). These outcomes demonstrate that the HEArT POC system exhibits the same quantitative accuracy as the clinical-standard ELISA technique and can effectively detect biomarkers in complex samples. Subsequently, we compared the MI_sig_ levels calculated by the HEArT system in AMI patients (*n* = 45) and non-AMI individuals (*n* = 132) (Fig. 6f). The average MI_sig_ of AMI samples (2.62 ± 2.73) was significantly higher than that of individuals without AMI (–4.35 ± 2.37, *P* < 0.0001). Based on the cut-off value obtained in Fig. 4f, an accurate AMI screening of 91.0% (95% CI: 86.8%–95.2%) was achieved, with a sensitivity of 80.0% (95% CI: 74.1%–85.9%) and a specificity of 94.7% (95% CI: 91.4%–98.0%) (Fig. 6g). Furthermore, we compared the CVD identification results given by the HEArT POC device with the clinical diagnosis results. HEArT achieved the identification of non-CVD individuals, SCHD patients, arrhythmia patients, CHF patients, and AMI patients with an accuracy of 90.6%, 83.1%, 50.0%, 60.0%, and 80.0%, respectively, and an overall accuracy of 77.4% (95% CI: 71.2%–83.6%) was obtained for multiclass CVD diagnosis, as summarized by a confusion matrix (Fig. 6h). The accuracy would increase to 92.7% (95% CI: 88.9%–96.5%) when the top two probabilities are taken into account. In addition, we also challenged the longitudinal monitoring of the HEArT POC system for patients after PCI treatment. We monitored the changes of MI_sig_ within 168 h after treatment in five AMI patients through HEArT and summarized them in Fig. 6i. Compared with the MI_sig_ levels before PCI treatment, the MI_sig_ values in AMI patients after PCI treatment were significantly lower, indicating alleviated myocardial injury after the operation. The above results demonstrate the excellent clinical applicability of the HEArT POC system in the fields of risk assessment, auxiliary diagnosis, and prognosis monitoring of CVD patients.

Among the assays designed for biomarker diagnosis, especially in the field of electrical biosensing technology, HEArT stands out for its high sensitivity and accuracy in clinical sample profiling (Table S4). With a cost of <$2 per test and a detection duration within 15 min, HEArT offers a feasible approach for the development of POC technology and the healthcare management of CVDs (Fig. 6j).

## DISCUSSION

Simultaneous profiling of multiple protein characteristics and their changes can provide important clinical guidance for the rapid screening and therapy strategies of CVD patients. However, the trace levels of biomarkers in plasma and the complex physiological environment pose challenges to the development of *in vitro* diagnostic assays. Herein, a HEArT POC assay was described to identify and analyze multiple MIBs in plasma samples for rapid and accurate screening and clinical management of CVD patients. The phospholipid self-assembly functionalized sensory interface exhibits excellent electrical properties and is designed to counteract the Debye screening effect caused by high ionic strength in the blood and reduce the non-specific adsorption of biomolecules, thus enabling the direct detection of protein signatures in freshly collected plasma samples. In addition, the phospholipid self-assembled layer can remain stable at 4°C for 7 d and is not affected by high ionic strength or water absorption swelling, thus showing considerable application potential among various surface modification strategies (Table S5). The HEArT biosensor array constructed by us can achieve precise detection of pg/mL level MIBs in plasma, with quantitative accuracy comparable to ELISA. Our assay can provide diagnosis results within 15 min, indicating potential applications in the field of POC detection.

We then explored the clinical applicability of HEArT. Through the quantitative analysis of blood samples of beagles with the AMI model at different time points, HEArT could report changes in MIBs within 15–90 min after AMI occurrence, and the detection time window is more than 30 min earlier compared with biochemical assays. By developing an interpretable data algorithm, HEArT was able to conduct a risk assessment of myocardial injury and accurately identify CVDs in clinical patients with chest pain, as well as analyze the specific relationship between biomarkers and CVD types. The continuous recording of MIB levels of AMI patients presented a significant correlation between marker characteristics and treatment response, reflecting the variations in risk of myocardial injury before and after PCI treatment, realizing the clinical judgment of MIRI, and demonstrating the potential of HEArT in efficacy evaluation, risk assessment, and prognosis monitoring.

In summary, we present the development and performance of a highly sensitive and clinically feasible HEArT POC technique for MIB profiling in human serum samples. HEArT could report the variations of protein signatures at pg/mL levels, classify CVDs by adapting an LDA algorithm, and monitor the prognosis of AMI patients. With its portability, high sensitivity, minimal invasiveness, and short duration of measurement, HEArT can be operated by untrained personnel and has the potential to be applied in standard clinical practice for the diagnosis and prognosis of CVDs.

### Limitations of the study

Despite the above promising outcomes, several limitations need to be addressed in future studies. In the current work, HEArT relies primarily on three biomarkers (cTnI, Myo, and CK-MB) for early screening of AMI, and was restricted by the insufficient amount of animal samples. Some non-cardiogenic markers, such as C-reactive protein, tumor necrosis factor-α, as well as miRNAs, have been proven to appear earlier in human plasma at the onset of AMI [59–63]. Expanding both markers and samples, as well as comprehensively analyzing the correlations of markers, are expected to further advance the diagnostic window of AMI. As for CVD classification, due to the focus on AMI identification in our current work, the classification accuracy for other CVD diseases by HEArT is relatively low, limited by the selection of biomarkers. In future works, it would be meaningful to develop a detection platform for CVD screening. Tests of other biomarkers, as well as clinical indicators such as heart rate, blood oxygen, and blood pressure, can be incorporated for this purpose. Though HEArT POC detection is superior in terms of LOD, detection time, multiplex detection, and instrument dependence, the accuracy and stability of HEArT for ultra-trace samples (<50 μL) need to be improved. Introducing artificial intelligence algorithms or self-calibration strategies might be an effective approach to solving the above problem. Also, we plan to further broaden the impact of the current work. First, we could expand the training cohort and optimize the parameters of the LDA algorithm to further improve the accuracy of CVD identification. Second, motivated by HEArT’s high sensitivity, we could explore the molecular mechanisms of CVDs and the relationship between biomarkers at a microscopic level. Third, although we have verified the application of this POC system in the management of CVDs, a similar platform can also be used for liquid biopsy, including blood, urine, and saliva, to expand the scenarios of personalized medicine and intelligent healthcare.

## METHODS AND MATERIALS

### Materials and reagents

Ethanol, chloroform, acetone, glutaraldehyde (25.0%), methanol, iron(III) chloride (FeCl_3_, >99.0%), and polyvinyl butyral were purchased from Sinopharm Chemical Reagent Co., Ltd. Polymethyl methacrylate (PMMA), OTES, and APTES were obtained from Aladdin. FBS and PBS (0.1 mM, 1 mM, and 10 mM, pH 7.4) were bought from Shanghai Yuanye Bio-Technology Co., Ltd. 1,2-Dihexadecanoyl-rac-glycero-3-phosphocholine (DPPC) was obtained from Suzhou Highfine Biotech Co., Ltd. Myocardial injury biomarkers CK-MB, cTnI, Myo, pro-BNP, D-dimer, and the corresponding antibody molecules anti-CK-MB, anti-cTnI, anti-Myo, anti-pro-BNP, and anti-D-dimer were purchased from Dafeng Biotechnology Co., Ltd. Detailed information on these proteins are listed in Table S6.

### Fabrication of MPI FET biosensors

The fabrication of MPI FET biosensors involves three steps, including SAM modification, antibody immobilization, and assembly of a phospholipid monolayer. Before surface modification, the exposed IGZO channels were washed three times with ethanol to remove surface contaminants. Subsequently, the MPI FET was immersed in APTES/OTES (5% ethanol, APTES 5 vol%, 10 vol%, 20 vol%, 33 vol%, 50 vol%, 66 vol%, 100 vol%, respectively) solution for 2 h to construct a SAM with reactive amine groups and a hydrophobic octadecyl chain. The MPI FET was placed onto a heating plate for 1 h at 120°C to strengthen the silanization of APTES and OTES. For antibody immobilization, the MPI FET was immersed in glutaraldehyde (2% PBS) for 1 h and incubated with antibody solution (10 μg/mL) overnight at 4°C. After washing with deionized water three times and drying with nitrogen, the sensing surface of MPI FET was dripped with vesicle solution and incubated for 5 h at room temperature to self-assemble a dense, homogeneous monolayer of phospholipids.

### Detection of MIBs in serum samples

To evaluate the contents of biomarkers in serum samples, HEArT biosensors were incubated with serum samples for 5 min at 30°C. Subsequently, the electrical responses were monitored by the transfer characteristic curves. The current responses (100 × Δ*I*/*I*_o_) of each serum sample were calculated from the ratio of the current change (Δ*I* = *I*_d_ − *I*_o_) and initial current (*I*_o_), respectively.

### Statistics analysis

The statistical analyses of this study were processed by GraphPad Prism 8 and Origin 9.6. The LDA algorithm was performed by SPSS 26.0. Statistical details for each experiment are shown in the figure legends.

### Ethical statements

The research was approved by the Ethics Committee of Renmin Hospital of Wuhan University (WDRY2022-K257, WHU-LFMD-2024 047). All the participants in this research provided signed informed consent forms before participating. The investigators strictly followed the implementation protocol approved by the Ethics Committee, and the implementation process was in accordance with the principles of the NMPA/GCP. Data organization and manuscript writing follow the TRIPOD/STARD guidelines.

## Supplementary Material

nwag156_Supplemental_File
