# Supplementary material for: A transistor-based point-of-care assay with lipid-capped sensory interface for clinical profiling of cardiovascular diseases
Source: Natl Sci Rev. 2026 Mar 11;13(8):nwag156. doi: 10.1093/nsr/nwag156 (PMC13131221; doi:10.1093/nsr/nwag156)
Supplement: nwag156_Supplemental_File [file nwag156_supplemental_file.pdf]

Supporting Information

A transistor-based point-of-care assay with lipid-capped sensory interface for clinical profiling of cardiovascular diseases

Duo Chen[1,†], Nan Song[1,†], Yun Zhang[1,†], Fuding Guo[1,5], Liping Zhou[1], Cunlan Guo[1], Xidong Duan[3], Dacheng Wei[4], Weihong Tan[2], Lilei Yu[1,*], Yanbing Yang[1,*], Quan Yuan[1,*]

[1]College of Chemistry and Molecular Sciences, Key Laboratory of Biomedical Polymers of Ministry of Education, Institute of Molecular Medicine, Department of Cardiology, Renmin Hospital of Wuhan University, State Key Laboratory of Metabolism and Regulation in Complex Organisms, Taikang Center for Life and Medical Sciences, Hubei Key Laboratory of Autonomic Nervous System Modulation, Cardiac Autonomic Nervous System Research Center of Wuhan University, Wuhan University, Wuhan 430072, China;

[2]Key Laboratory of Zhejiang Province for Aptamers and Theranostics, Zhejiang Cancer Hospital, Hangzhou Institute of Medicine (HIM), Chinese Academy of Sciences, Hangzhou 310022, China;

[3]Hunan Key Laboratory of Two-Dimensional Materials, State Key Laboratory of Chemo and Biosensing, College of Chemistry and Chemical Engineering, Hunan University, Changsha 410082, China;

[4]State Key Laboratory of Molecular Engineering of Polymers, Laboratory of Molecular Materials and Devices, Department of Macromolecular Science, Fudan University, Shanghai 200433, China;

[5]Key Laboratory of Cardiovascular Disease of Yunnan Province, Clinical Medicine Center for Cardiovascular Disease of Yunnan Province, Department of Cardiology, Yan'an Affiliated Hospital of Kunming Medical University, Kunming 650000, China

*Corresponding authors. Emails: yuanquan@whu.edu.cn; yangyanbing@whu.edu.cn; lileiyu@whu.edu.cn

[†]**Equally contributed to this work.**

47 supplementary figures, 6 supplementary tables, and 3 supplementary videos

Contents

Supplementary methods

Supplementary figures

Figure S1. SEM image of a cross-sectional IGZO channel.
Figure S2. Transfer characteristic curves of MPI FET.
Figure S3. Confocal laser scanning microscopy images of MPI FET surface.
Figure S4. ATR-FTIR spectra of MPI FET and IGZO FET.
Figure S5. XPS spectra of MPI FET and IGZO FET.
Figure S6. AFM characterization of the phospholipid monolayer.
Figure S7. Interface hydrophilicity of IGZO channel.
Figure S8. AFM characterization of MPI FET and IGZO FET.
Figure S9. Optimization of measurement conditions.
Figure S10. Detection capability of IGZO FET.
Figure S11. Exploration of vesicle incubation time.
Figure S12. Anti-biofouling capability of IGZO FET.
Figure S13. Interface hydrophilicity of MPI FET surface.
Figure S14. Calibration curves of MPI FET toward cTnI in FBS with different SAM modifications.
Figure S15. Verification of the detection performance at the pg/ml level.
Figure S16. Stability of MPI FETs for long-term storage.
Figure S17. N atoms distribution above MPI FET interfaces.
Figure S18. The molar concentration of ions at the sensory interface in 0.5 M electrolyte.
Figure S19. The molar concentration of ions at the sensory interface in 1.0 M electrolyte.
Figure S20. The molar concentration of ions at the sensory interface in 1.5 M electrolyte.
Figure S21. Ion distribution at the sensory interface of MPI FET and IGZO FET.
Figure S22. Screening factors at the sensory interface of MPI FET and IGZO FET.
Figure S23. Experimental evidence of the interface screening effect.
Figure S24. Comparison of screening factors under different DPPC coverage.
Figure S25. Ultraviolet-visible spectroscopy after non-specific adsorption.
Figure S26. QCM evidence of the anti-fouling capabilities.
Figure S27. Multi-channel HEArT biosensing array.
Figure S28. Crosstalk verification of HEArT biosensing array.
Figure S29. Detection capability of HEArT biosensing array.
Figure S30. Quantitative accuracy of HEArT biosensing array.
Figure S31. Specificity verification of HEArT biosensing array.
Figure S32. Evaluation of cTnI in the serum of a control beagle.
Figure S33. Evaluation of cTnI in the serum of AMI beagles.
Figure S34. Evaluation of Myo in the serum of beagles modeling.
Figure S35. Evaluation of CK-MB in the serum of beagles modeling.
Figure S36. Evaluation of pro-BNP in the serum of beagles modeling.
Figure S37. Evaluation of D-Dimer in the serum of beagles modeling.
Figure S38. Discriminate AMI patients and non-AMI patients *via* SUM of MIB responses.

Figure S39. PRC curves and average precision for AMI identification.
Figure S40. Sensitivity and specificity of AMI diagnosis *via* MIB signatures.
Figure S41. Cardiovascular disease classification results *via* single MIB signatures and SUM.
Figure S42. Accuracy of AMI diagnosis and CVD classification via different MIB signature combinations.
Figure S43. Longitudinal monitoring of 18 AMI patients undergoing PCI treatment.
Figure S44. Clinical examination results of patients with poor prognosis.
Figure S45. Photos of the HEArT POC devices and their internal structure.
Figure S46. The repeatability of the HEArT POC array.
Figure S47. Verification and optimization of filtration performance for serum extraction.

Supplementary tables

Table S1. The detection performance of the MPI FET biosensor toward cTnI, Myo, CK-MB, pro-BNP, and D-Dimer.
Table S2. Summary of the weight ratios and coefficient of variation through 5-fold cross-validation in the training set.
Table S3. Recovery of HEArT biosensing array.
Table S4. Comparison of the sensing performance of reported POC devices.
Table S5. Summary and Comparison of Electrode Surface Modification Strategies and Detection Performance.
Table S6. List of proteins used in this work.

Supplementary videos

Video S1. The HEArT POC system detects a clinical serum sample from an AMI patient *via* a personal computer.
Video S2. The HEArT POC system detects a clinical serum sample from a non-CVD individual *via* a smartphone.
Video S3. The HEArT POC system detects a clinical serum sample from an AMI patient *via* a smartphone.

Supplementary methods

Instrument and characterization

The Cr/Au electrode array of FET was patterned by ultra-violet (UV) lithography (ABM Inc., America) and deposited by a thermal evaporator system (Jiashuo JSD-300, China). The IGZO channel materials were deposited by a radio frequency sputtering system with the assistance of mask templates (Jiashuo JSD300-II, China). The sensing area of the FET biosensor was exposed by an electron beam lithography system (EBL, JSM-6510, Japan). The microscopy images of the IGZO sensing electrodes were obtained by an optical microscope (Olympus BX53MRF-S). The modification of antibody molecules on the IGZO surface was characterized by XPS (ESCAI AB250Xi, USA) and ATR-FTIR (FTIR5700, USA). AFM (Park nx10, Korea) characterized the surface morphology of the sensory interface. The electrical characteristics of FET devices were measured by a digital source meter (Agilent B2902A, USA) connected to a probe station.

Fabrication of FET arrays

The FET arrays were fabricated by several conventional processes of semiconductor device manufacturing. The interdigital Cr/Au (10/30 nm) source, drain, and gate electrodes were constructed via thermal evaporation with the protection of a mask template after being patterned by photolithography. The channel length and width were 480 μm and 500 μm, respectively. The IGZO channel materials were deposited by magnetron sputtering between the interdigitate source and drain electrodes. PMMA was spin-coated on the surface of IGZO FET as the protective layer. The sensing area of the IGZO FET was exposed by an electron beam lithography system. Ag/AgCl reference electrodes were fabricated by sequentially dip-coating silver colloid on the gate electrode, heating at 75 °C for 15 min to evaporate the solvent, dropping 0.1 M $FeCl_3$ solution on the silver colloid, and reacting for 4 min to form an AgCl layer. The surface of AgCl electrodes was then immersed in the polyvinyl butyral-methanol solution for 3 h at room temperature to form a protective layer.

Preparation of DPPC vesicles

The vesicles were prepared from a chloroform stock of DPPC (50 mg/mL). The chloroform was removed by rotary evaporation. The dry lipids were rehydrated at 45 °C for 3 h in deionized water, and the total vesicle concentration was 0.5 mg/mL. The obtained vesicle solutions were extruded 21 times through a 200-nm-diameter polycarbonate filter membrane (Avanti Polar Lipids Inc., USA) to prepare uniformly sized vesicles.

Detection of MIBs by HEArT

The sensing regions of HEArT were dripped with varying concentrations (10^{-16}–10^{-6} g/mL) of MIB solution and incubated for 5 mins at room temperature. Then, the HEArT arrays were rinsed with PBS three times to remove any unbound proteins. To evaluate the incidence of target protein conjugation, the calibrated I_d-V_g curves before and after incubation were recorded at V_d

= 200 mV.

The LOD in this study was calculated using the 3-fold signal-to-noise ratio method (3σ/S). The specific steps are: determining the current response value of blank serum samples and calculating the standard deviation (σ), drawing the response curve of target substances at different concentrations and calculating the curve slope (S), and then calculating the LOD according to the formula LOD = 3σ/S.

Molecular Dynamics Simulations

In order to study the distribution of ions on phospholipids and IGZO surfaces in KCl solutions, MD simulation was performed using the LAMMPS software package [64]. We used the Lipid14 force field to model phospholipid [65]. The initial configuration of phospholipids was generated by CHARMM-GUI using Membrane Builder software [66]. The water molecules were modeled using the TIP3P model, and the remaining atoms were modeled using the CHARMM force field. The SHAKE algorithm is used to fix the bond length and Angle in water molecules, and the long-term electrostatic interactions are calculated using PPPM. For all simulation systems, periodic boundaries are applied in the x and y directions, and aperiodic boundaries are applied in the z direction. Prior to the formal simulation, each system underwent energy minimization to keep the model stable. All MD simulations in this paper are performed in an NVT ensemble at a temperature of 300 K. The time step for all MD simulations is set to 1 fs.

Animal modeling and sample collection

The research conforms to the Guide for the Care and Use of Laboratory Animals published by the US National Institutes of Health (NIH Publication No. 85-23, revised 1996). All animal studies were reviewed and approved by the animal experimental administration of Wuhan University. All the beagles were anesthetized with 30 mg/kg Na-pentobarbital and ventilated with room air by a positive pressure ventilator. At the end of each hour during the procedure, an additional dose of 2 mg/kg Na-pentobarbital was administered to maintain the dogs anesthetized. The core body temperature of the dogs was maintained at 36.5 ± 1.5 ºC by the heating pad. Five beagles were randomized into the control group (N = 1) and the myocardial infarction group (N = 4). The MI model was established by LAD treatment. The control group was treated with a sham operation. The serum samples from beagles were collected 15 min before LAD treatment and 0, 5, 10, 15, 30, 45, 60, 75, 90, 105, and 120 min after LAD treatment.

Clinical samples

The clinical serum samples used in this research were provided by Renmin Hospital of Wuhan University, including 113 samples from AMI patients, 159 samples from SCHD patients, 73 samples from arrhythmia patients, and 82 serum samples from healthy individuals. Coagulated samples can cause protein adsorption on blood clots; therefore, we require centrifugation within 2 hours after sample collection to avoid coagulation. 442 clinical samples were randomly

divided into the training set and the test set at a ratio of 60:40. All relevant ethical laws and regulations concerning human participants were complied with.

LDA algorithms

We designed a two-step LDA to perform AMI identification and CVD classification. The first LDA discriminated between AMI patients and non-AMI individuals. The second LDA classified different CVD types. Both LDAs were using 5 MIB signatures as inputs. In the first LDA, we determined the optimal weights of each biomarker by logistic regression and calculated the MI_{sig} for each individual. In the second LDA, we used a max-wins voting strategy to determine the most possible CVD types. All LDA algorithms (1 for AMI identification and 6 for CVD classification) were optimized to achieve the best performance with the highest classification accuracy.

Before calculating the weights, we first standardized the current response values of the 5 biomarkers to eliminate the dimensional difference between different indicators. Furthermore, we calculated the within-class scatter matrix (S_v) and between-class scatter matrix (S_β) of each indicator data, and constructed the objective function $J(w) = w^T \times S_\beta \times w / w^T \times S_v \times w$ (where w is the weight vector). Subsequently, we normalized the weight vector of each indicator so that the sum of the absolute values of each weight is 100% to obtain the weight ratio of each indicator.

Supplementary figures

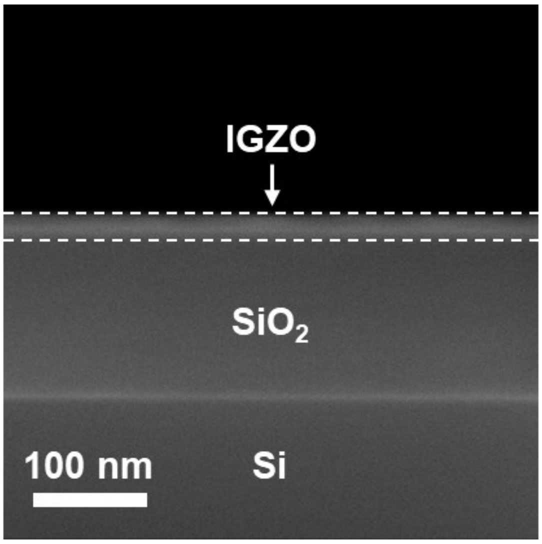

Figure S1. SEM image of cross-sectional IGZO channel. The thickness of the IGZO channel is about 25 nm.

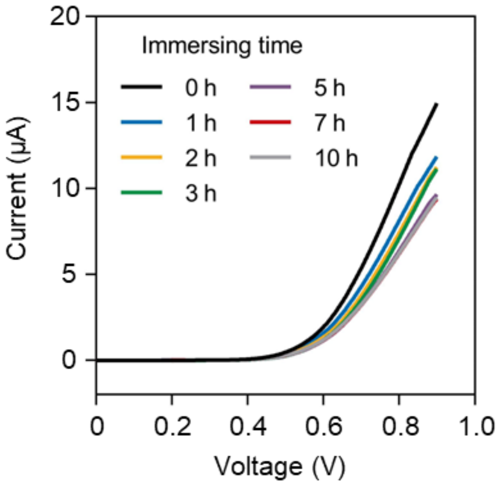

Figure S2. Transfer characteristic curves of MPI FET. The carrier mobility gradually decreased with the assembly of the phospholipid layer.

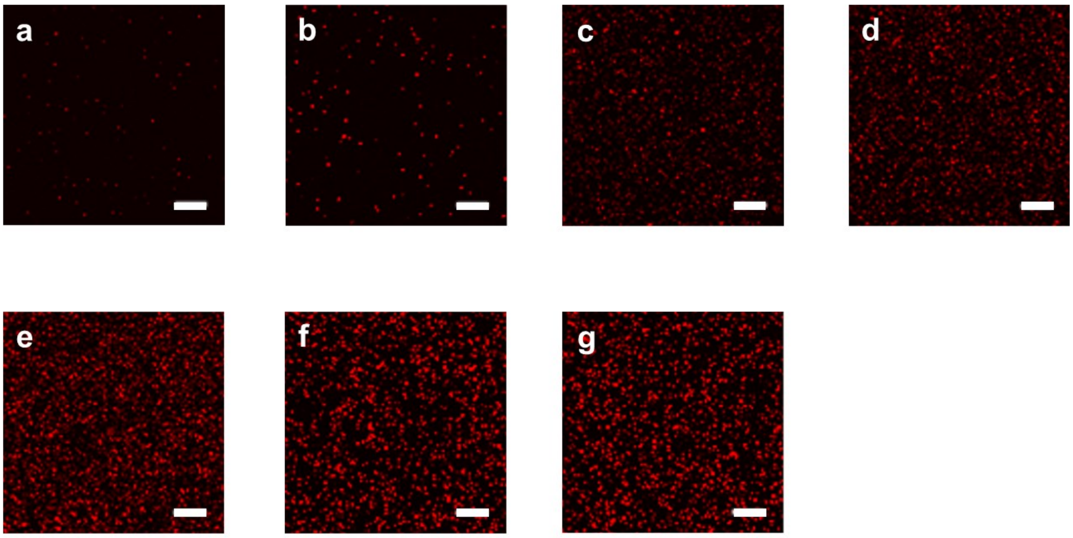

Figure S3. Confocal laser scanning microscopy images of MPI FET surface. MPI FETs were immersed in DPPC vesicle suspension for (**a**) 0 h, (**b**) 1 h, (**c**) 2 h, (**d**) 3 h, (**e**) 5 h, (**f**) 7 h, and (**g**) 10 h. The alkyl chain of DPPC was labeled by DiI. Scale bar: 10 nm.

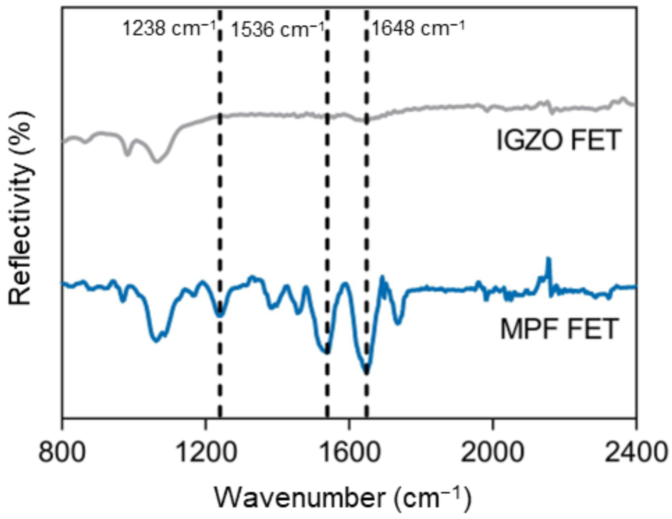

Figure S4. ATR-FTIR spectra of MPI FET and IGZO FET. The absorption peaks of phosphate skeleton (1238 cm^{-1}) and amide bond (1536 cm^{-1} and 1648 cm^{-1}) are labeled on the spectra.

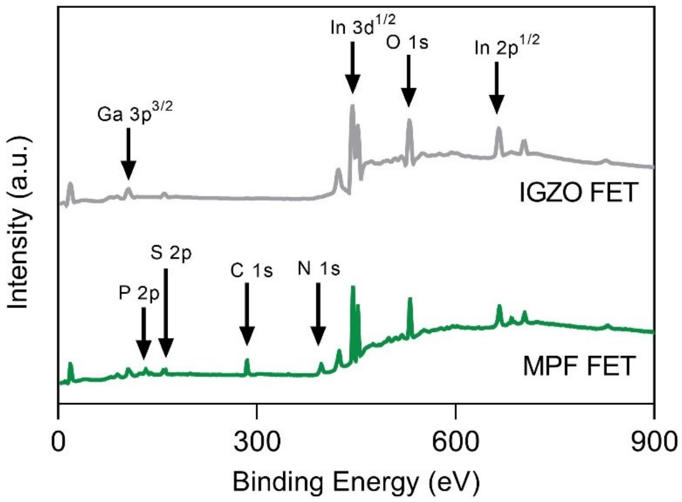

Figure S5. XPS spectra of MPI FET and IGZO FET. The characteristic peaks of C 1s (286.7 eV), N 1s (399.8 eV), S 2p (162.2 eV), and P 2p (132.3 eV) are labeled on the spectra.

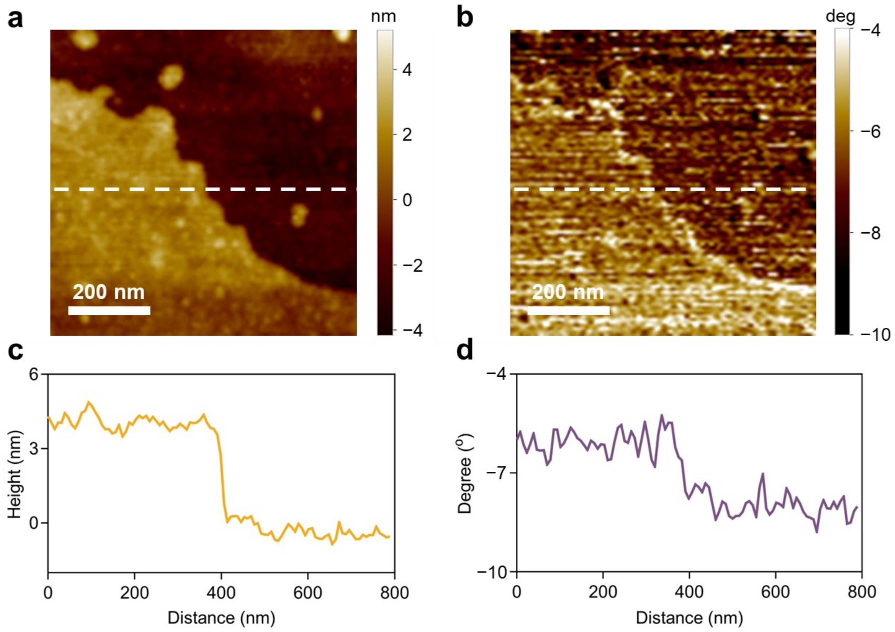

Figure S6. AFM characterization of the phospholipid monolayer. The (**a**) height image and (**c**) height profile indicate that the thickness of the phospholipid layer is about 4 nm. The (**b**) phase image and (**d**) degree profile present the boundaries of the phospholipid monolayer.

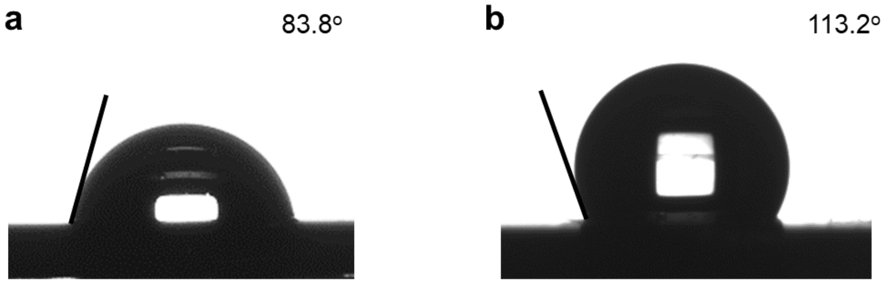

Figure S7. Interface hydrophilicity of IGZO channel. Water contact angles of IGZO channel (**a**) before and (**b**) after SAM modification.

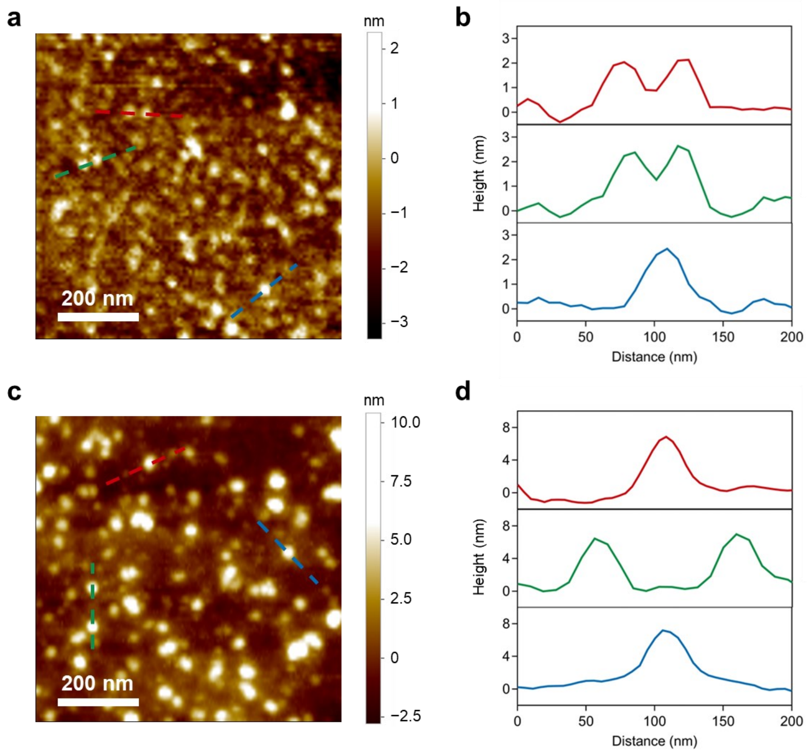

Figure S8. AFM characterization of MPI FET and IGZO FET. The (**a**, **c**) height image and (**b**, **d**) height profile of (**a**, **b**) MPI FET and (**c**, **d**) IGZO FET interface. The average height of cTnI antibodies on the surface of the MPI FET is ~ 2-3 nm. The average height of cTnI antibodies on the surface of the IGZO FET is ~ 6-8 nm.

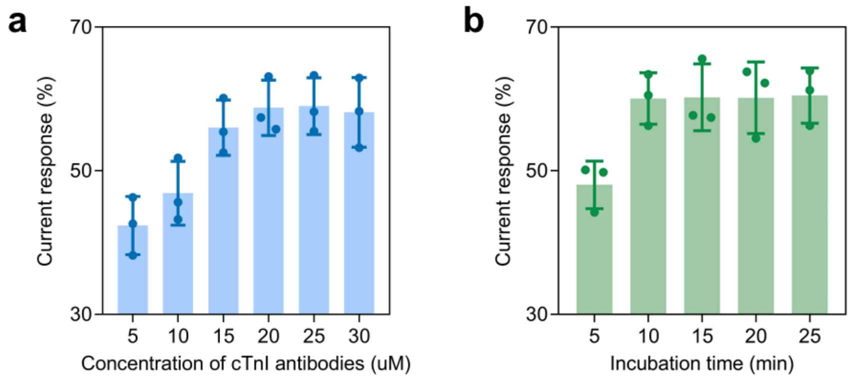

Figure S9. Optimization of measurement conditions. The current responses of MPI FET under different (**a**) antibody modification concentrations and (**b**) target-protein incubation time toward 1 ng/ml cTnI antigen (means ± s.d., n = 3). The antibody modification concentrations and target-protein incubation time were optimized at 20 ug/ml and 10 min, respectively.

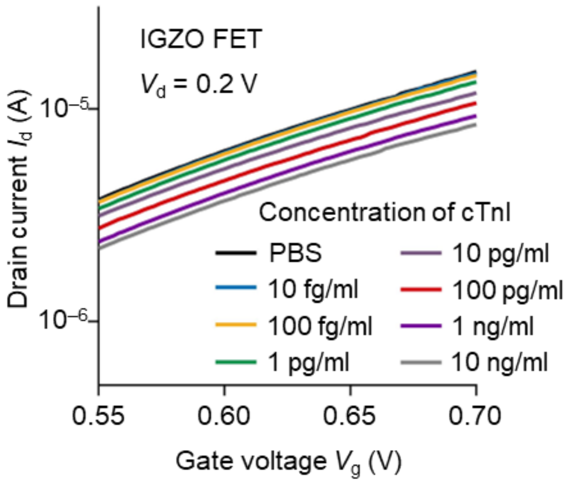

Figure S10. Detection capability of IGZO FET. I_d-V_g curves of IGZO FETs in response to different concentrations of cTnI. The source-drain voltage was set at 0.2 V.

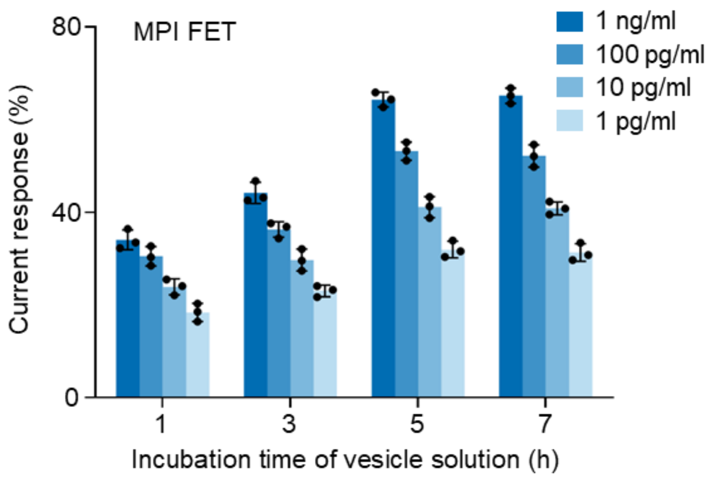

Figure S11. Exploration of vesicle incubation time. Current responses of MPI FET toward cTnI in FBS after different incubation times of vesicle solution (means ± s.d., n = 3).

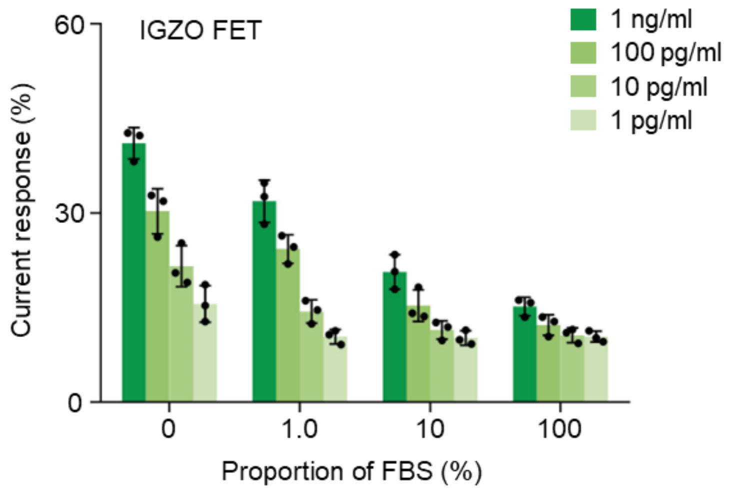

Figure S12. Anti-biofouling capability of IGZO FET. Current responses of MPI FET toward cTnI under different proportions of FBS (means ± s.d., n = 3).

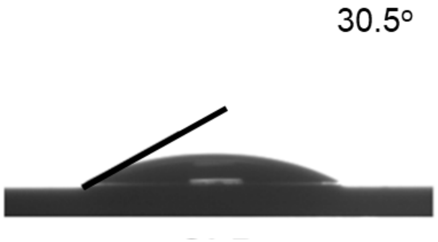

Figure S13. Interface hydrophilicity of MPI FET surface. Water contact angles of the self-assembled lipid-capped sensory interface. A hydrophilic surface is formed due to the presence of hydration layers.

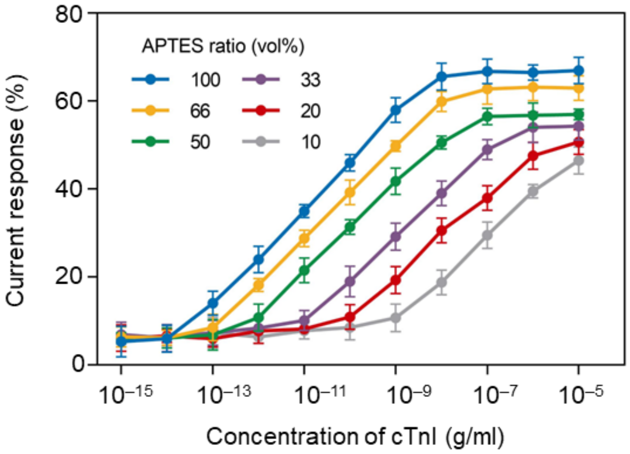

Figure S14. Calibration curves of MPI FET toward cTnI in FBS with different SAM modifications. Current responses of MPI FET modified with different hybrid SAMs toward cTnI are plotted (means ± s.d., n = 3). The hybrid SAMs were constructed by adjusting the ingredient ratios between APTES and OTES.

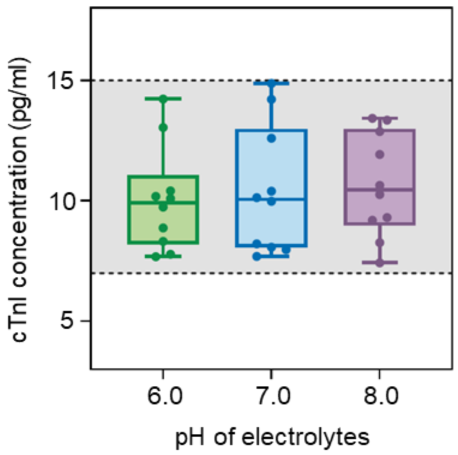

Figure S15. Verification of the detection performance at the pg/ml level. Recovery of MPI FET toward 10 pg/ml cTnI under different pH levels of electrolytes. The box plots show the median, interquartile range, and extreme values of 10 technical replicates. The recovery rate between 75% and 150% was labeled in gray.

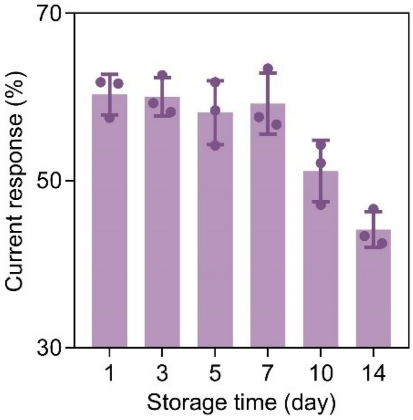

Figure S16. Stability of MPI FETs for long-term storage. The current responses of MPI FETs toward 1 ng/ml cTnI after different storage times at 4 °C (means ± s.d., n = 3).

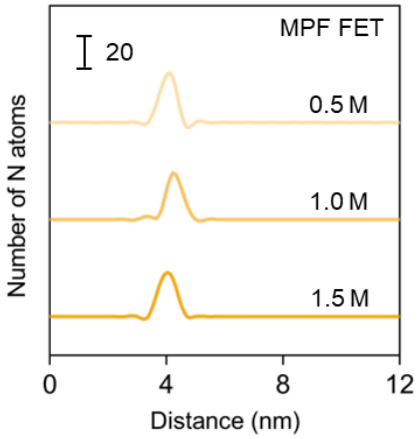

Figure S17. N atoms distribution above MPI FET interfaces. The relationship between the number of N atoms and the distance to the sensory interface of MPI FET under different electrolytes.

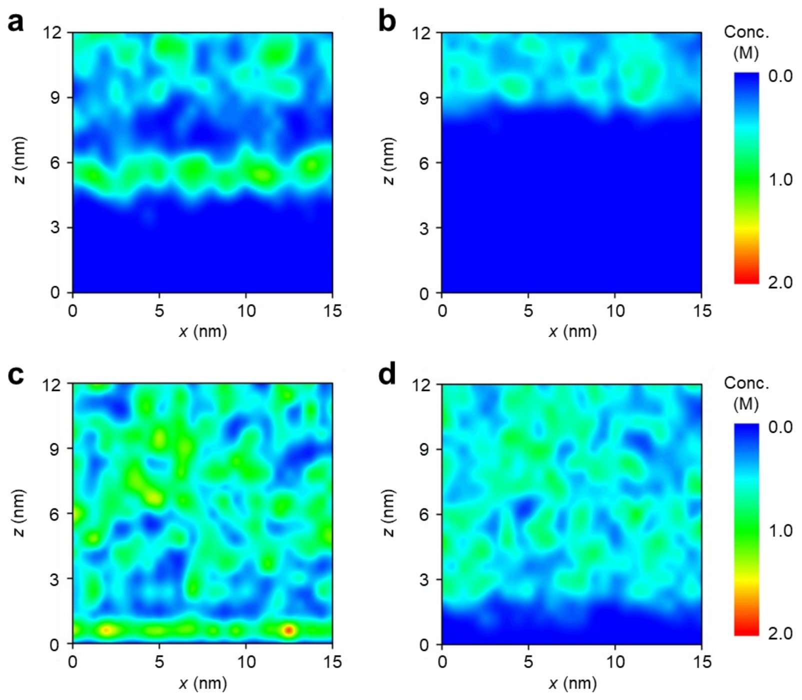

Figure S18. The molar concentration of ions at the sensory interface in 0.5 M electrolyte. Comparison of (**a**, **c**) K^+ distribution and (**b**, **d**) Cl^- distribution above the sensory interface of (**a**, **b**) MPI FET and (**c**, **d**) IGZO FET.

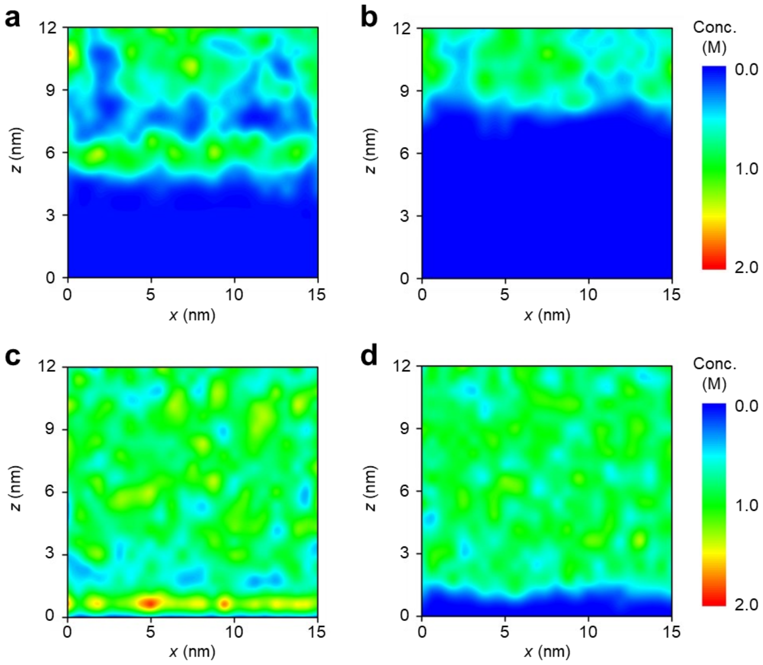

Figure S19. The molar concentration of ions at the sensory interface in 1.0 M electrolyte. Comparison of (**a**, **c**) K^+ distribution and (**b**, **d**) Cl^- distribution above the sensory interface of (**a**, **b**) MPI FET and (**c**, **d**) IGZO FET.

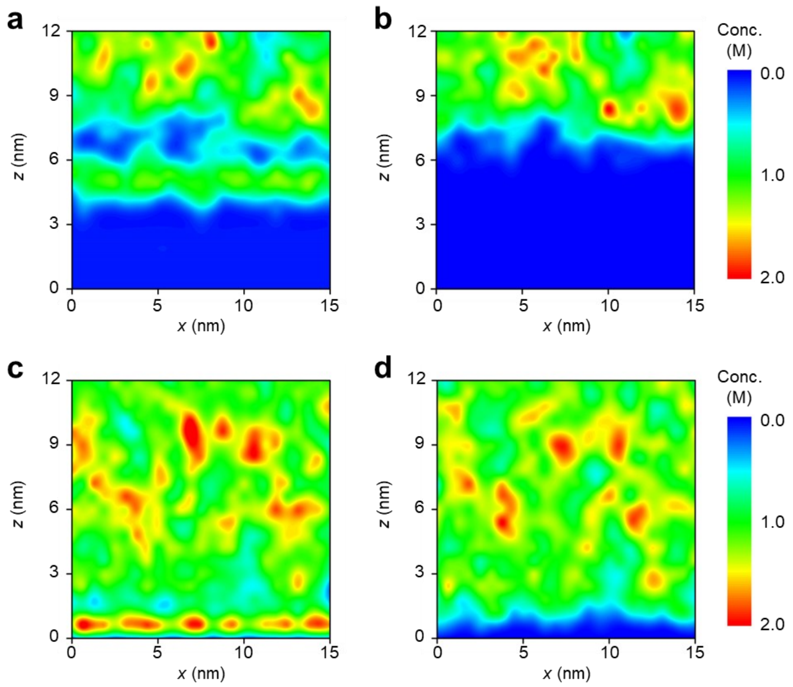

Figure S20. The molar concentration of ions at the sensory interface in 1.5 M electrolyte. Comparison of (**a**, **c**) K^+ distribution and (**b**, **d**) Cl^- distribution above the sensory interface of (**a**, **b**) MPI FET and (**c**, **d**) IGZO FET.

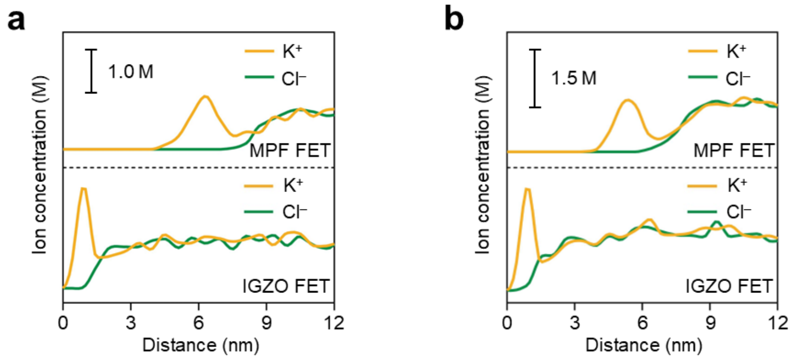

Figure S21. Ion distribution at the sensory interface of MPI FET and IGZO FET. Comparison of K^+ and Cl^- distribution above the sensory interface of MPI FET and IGZO FET in (**a**) 1.0 M and (**b**) 1.5 M KCl electrolyte.

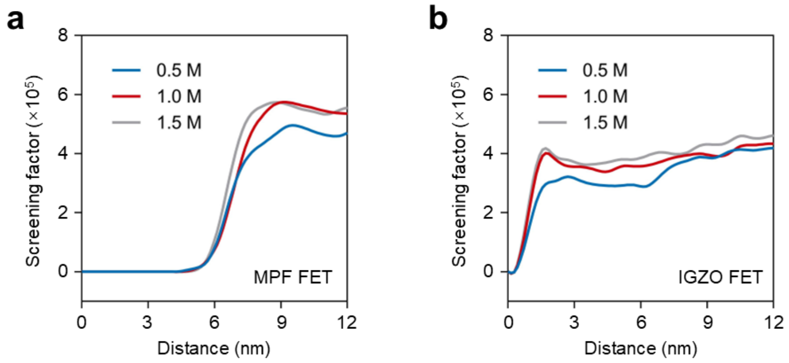

Figure S22. Screening factors at the sensory interface of MPI FET and IGZO FET. The relationship between the screening factor and the distance to the sensory interface of (**a**) MPI FET and (**b**) IGZO FET under different electrolytes.

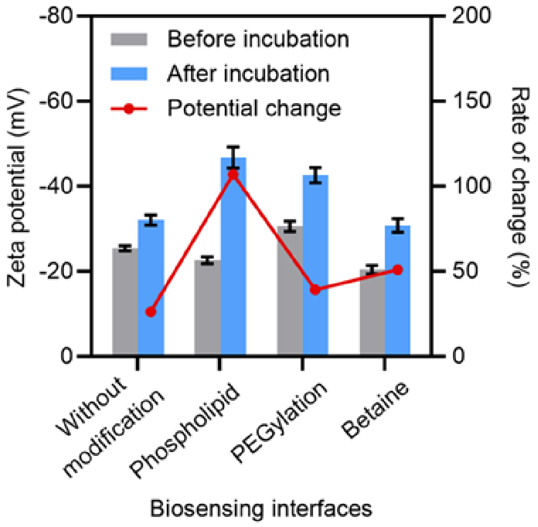

Figure S23. Experimental evidence of the interface screening effect. The changes in surface Zeta potential of different functionalized sensing interfaces before and after incubation with antigens.

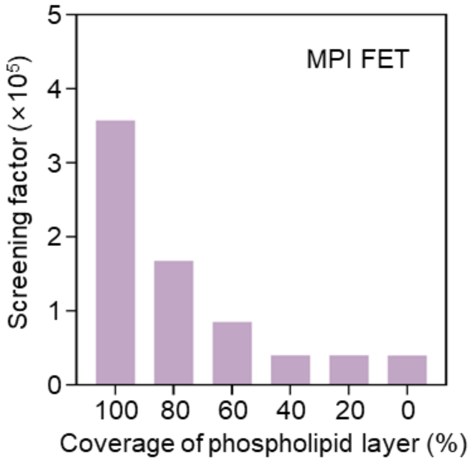

Figure S24. Comparison of screening factors under different DPPC coverage. Comparison of screening factors at 7 nm above the interface of MPI FET with different coverage rates of phospholipid layer. The electrolyte concentrations were set as 1.0 M.

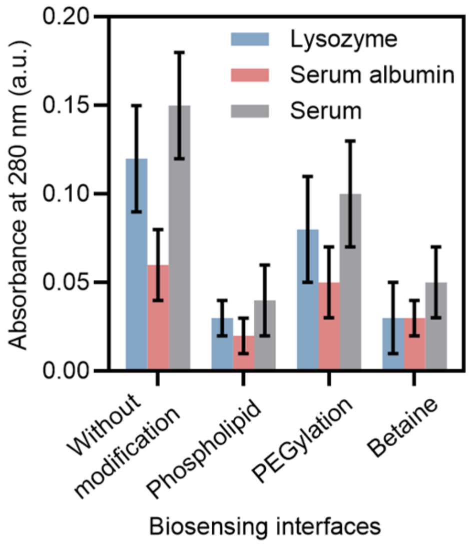

Figure S25. Ultraviolet-visible spectroscopy after non-specific adsorption. The absorbance at 280 nm of different functionalized sensing interfaces before and after incubation with lysozyme, serum albumin, and serum.

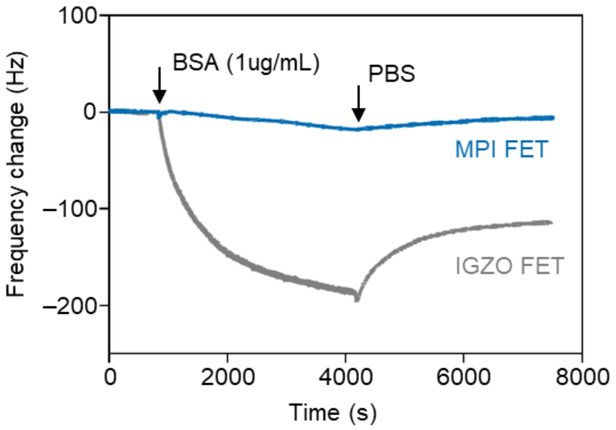

Figure S26. QCM evidence of the anti-fouling capabilities. QCM frequency shift of the BSA solution and PBS buffer on the interface of MPI FET (blue) and IGZO FET (grey).

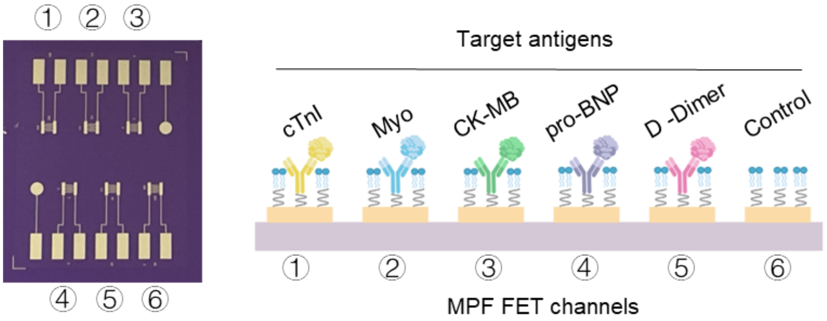

Figure S27. Multi-channel HEArT biosensing array. The schematic diagram of the HEArT biosensing array consists of 6 MPI FETs. The arrays were designed to simultaneously detect five MIBs (cTnI, Myo, CK-MB, BNP, and D-Dimer).

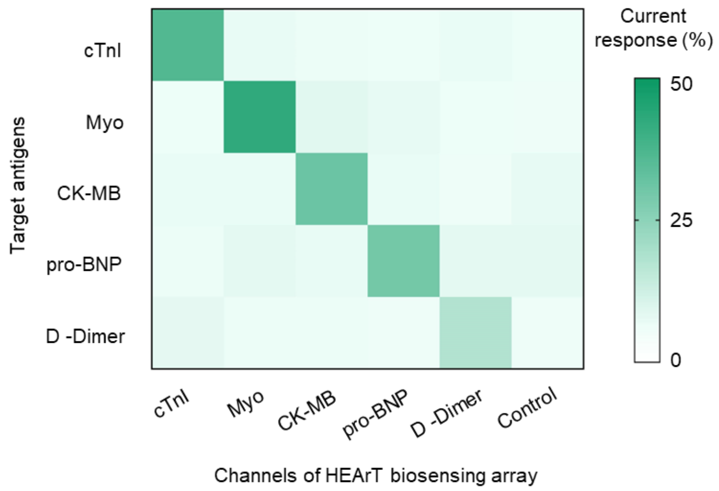

Figure S28. Crosstalk verification of HEArT biosensing array. The current responses of the multi-channel HEArT biosensing arrays toward different MIBs at concentrations of 1ng/ml. The color intensity indicates the level of current responses.

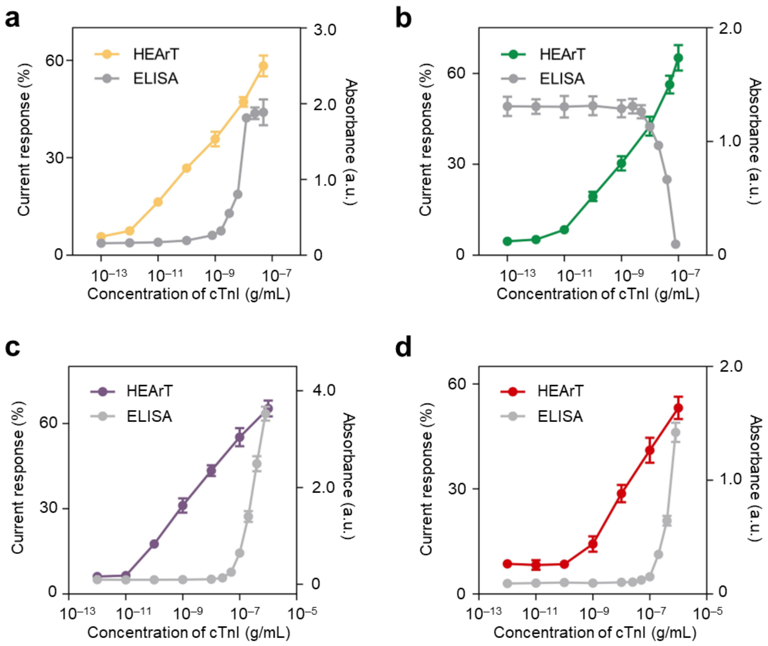

Figure S29. Detection capability of HEArT biosensing array. Comparison of detection capabilities of HEArT and ELISA assays targeting different concentrations of (**a**) Myo, (**b**) CK-MB, (**c**) pro-BNP, and (**d**) D-Dimer (means ± s.d., n = 3).

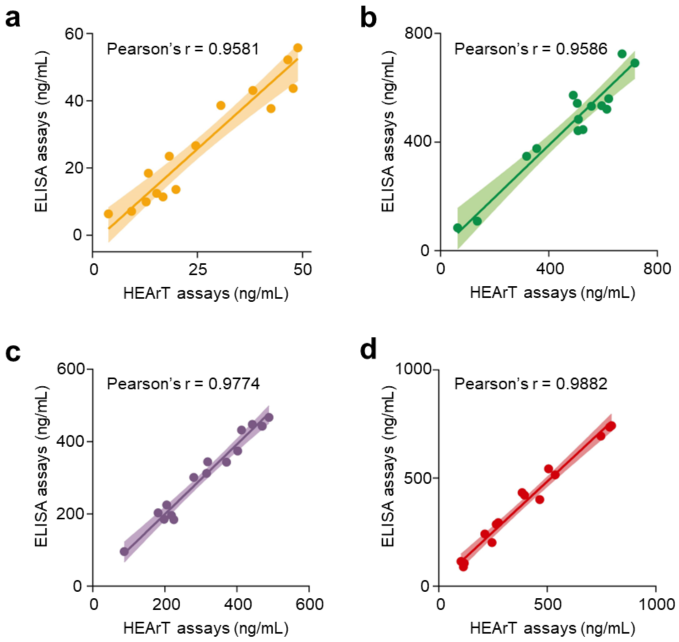

Figure S30. Quantitative accuracy of HEArT biosensing array. Correlation of the measurement results of HEArT and ELISA assays targeting (**a**) Myo (Pearson correlation coefficient, r = 0.9581), (**b**) CK-MB (Pearson correlation coefficient, r = 0.9586), (**c**) pro-BNP (Pearson correlation coefficient, r = 0.9774), and (**d**) D-Dimer (Pearson correlation coefficient, r = 0.9882).

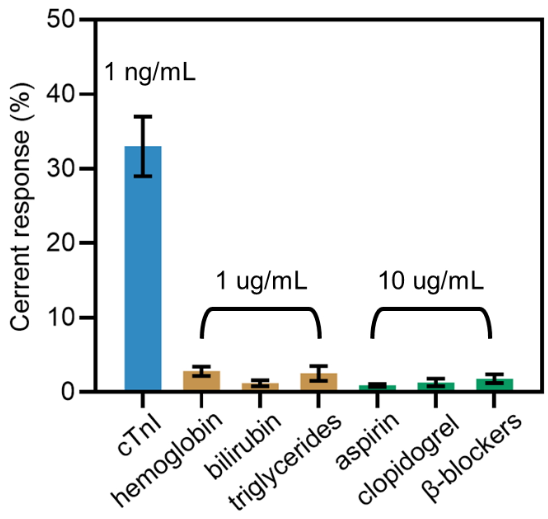

Figure S31. Specificity verification of HEArT biosensing array. The current responses of the MPI FET biosensor modified with cTnI antibody toward the cTnI antigen (1 ng/mL), hemoglobin (1 ug/mL), bilirubin (1 ug/mL), triglycerides (1 ug/mL), and common drugs (aspirin, clopidogrel, and β-blockers, 10 ug/mL).

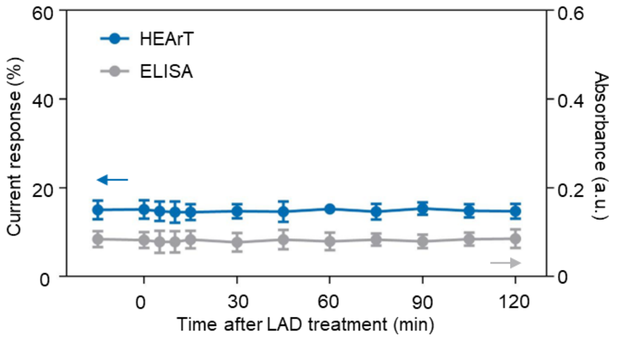

Figure S32. Evaluation of cTnI in the serum of a control beagle. Detection performance of HEArT and ELISA assays for the detection of cTnI in serum samples of beagles after sham operation (means ± s.d., n = 3).

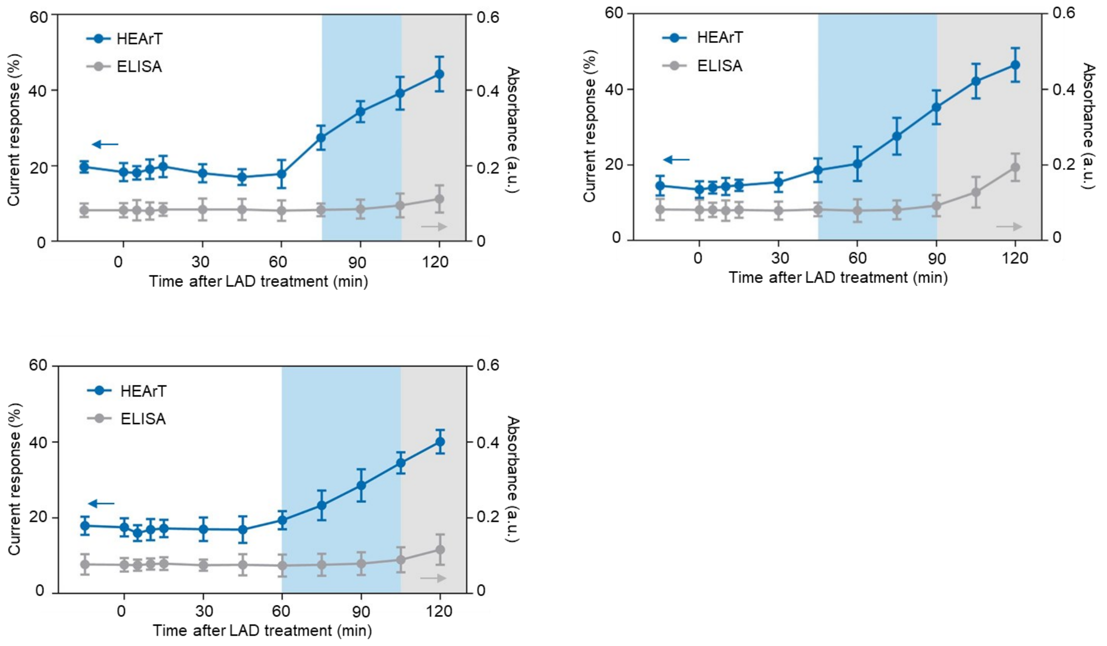

Figure S33. Evaluation of cTnI in the serum of AMI beagles. Detection performance of HEArT and ELISA assays for the detection of cTnI in serum samples of beagles after LAD treatment (means ± s.d., n = 3). The detection time window of HEArT is labeled in blue, while the detection time window of ELISA is labeled in gray.

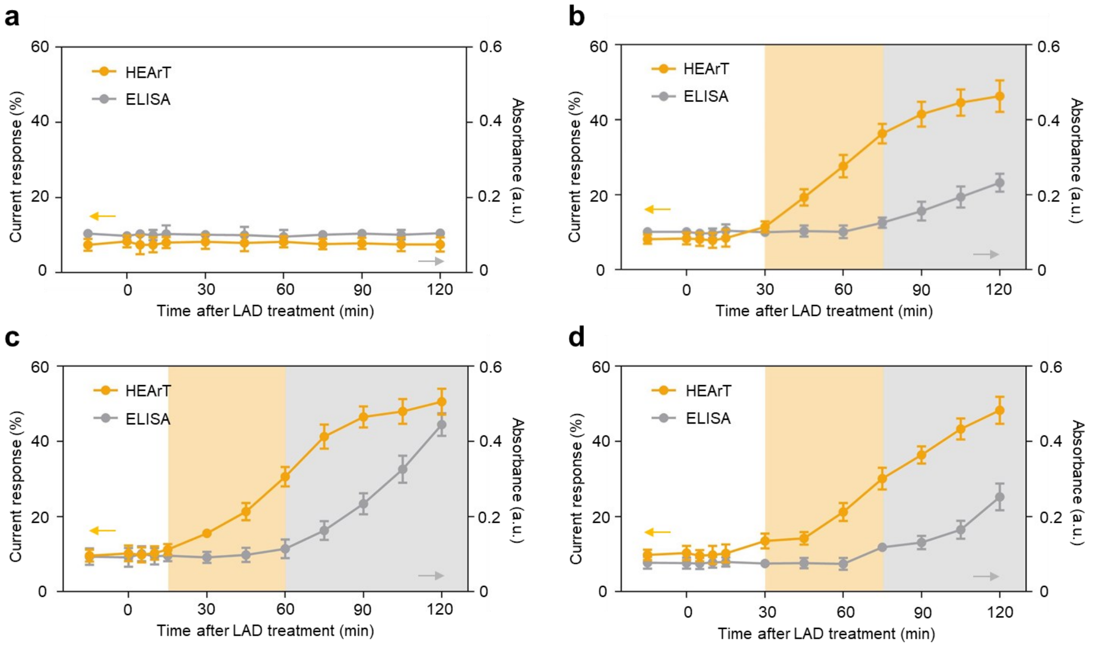

Figure S34. Evaluation of Myo in the serum of beagles modeling. Detection performance of HEArT and ELISA assays for the detection of Myo in serum samples of the (**a**) control beagle and (**b**, **c**, **d**) AMI beagles (means ± s.d., n = 3). The detection time window of HEArT is labeled in yellow, while the detection time window of ELISA is labeled in gray.

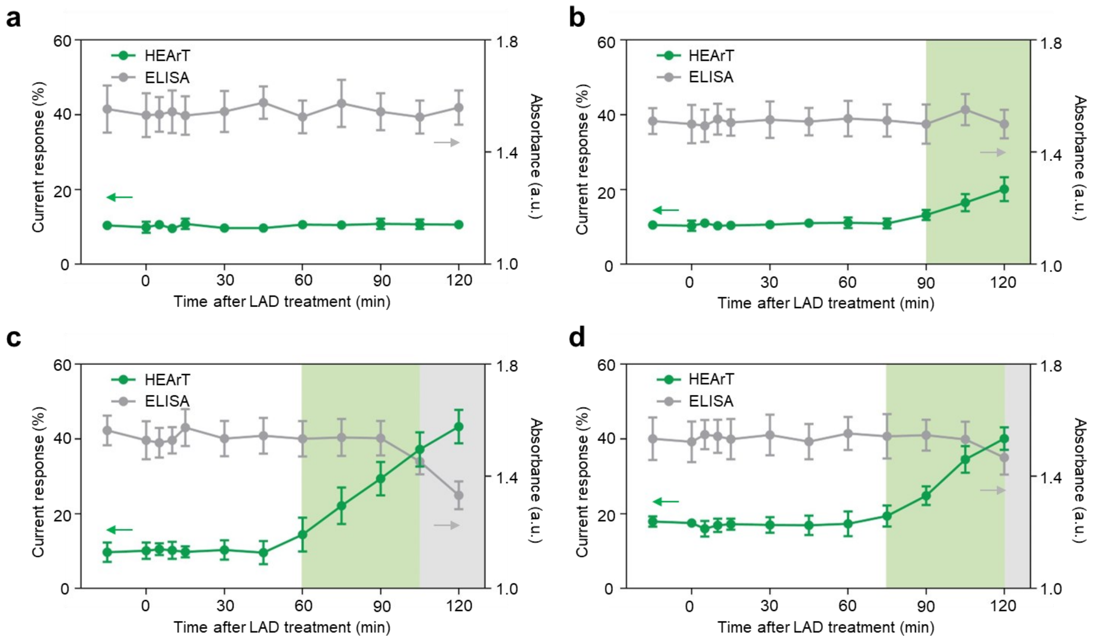

Figure S35. Evaluation of CK-MB in the serum of beagles modeling. Detection performance of HEArT and ELISA assays for the detection of CK-MB in serum samples of the (**a**) control beagle and (**b**, **c**, **d**) AMI beagles (means ± s.d., n = 3). The detection time window of HEArT is labeled in green, while the detection time window of ELISA is labeled in gray.

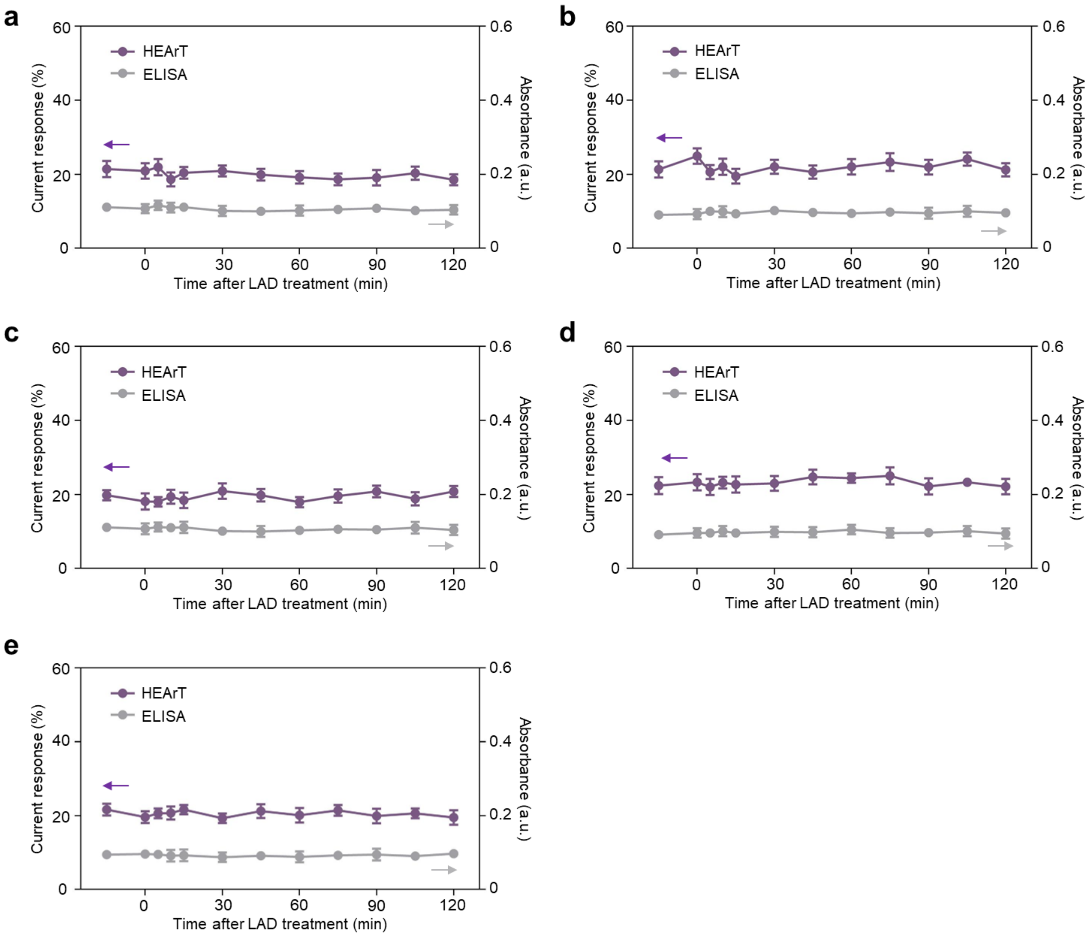

Figure S36. Evaluation of pro-BNP in the serum of beagles modeling. Detection performance of HEArT and ELISA assays for the detection of pro-BNP in serum samples of the (**a**) control beagle and (**b**, **c**, **d**, **e**) AMI beagles (means ± s.d., n = 3).

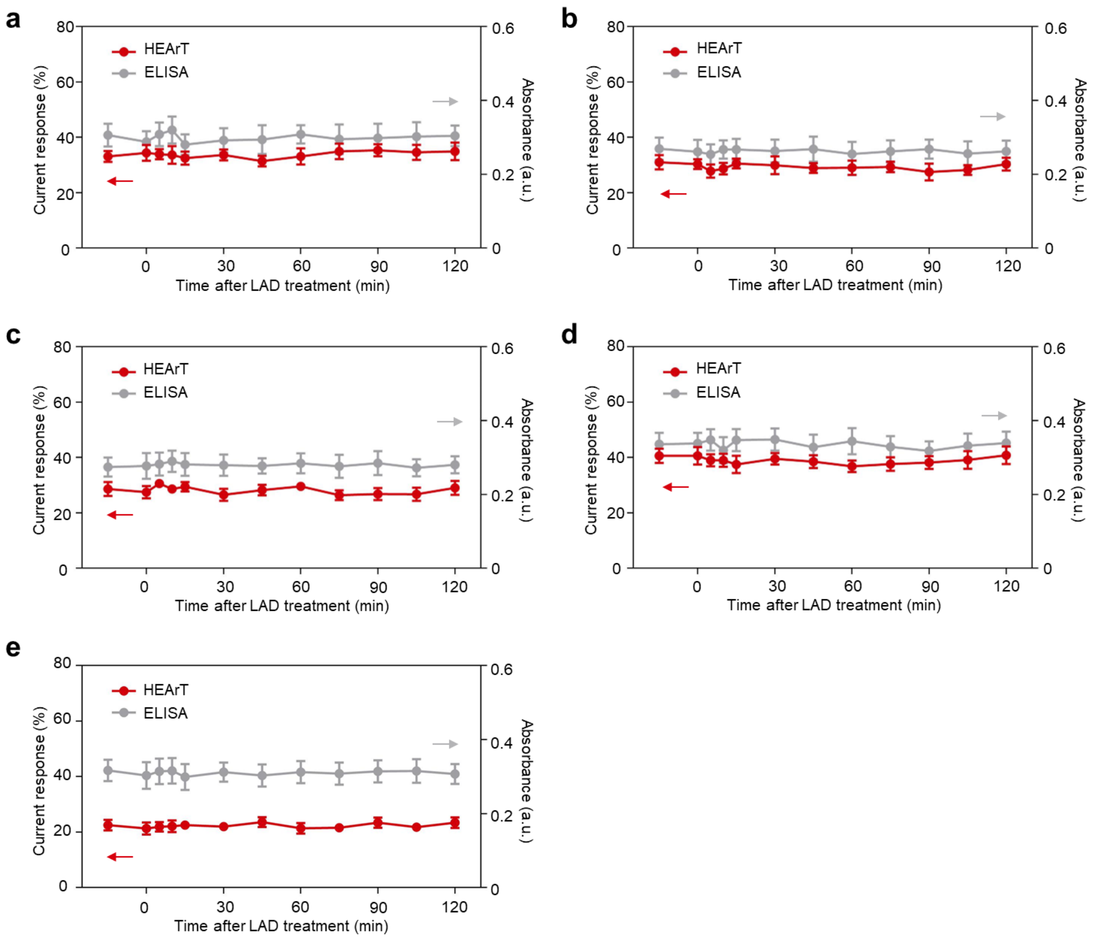

Figure S37. Evaluation of D-Dimer in the serum of beagles modeling. Detection performance of HEArT and ELISA assays for the detection of D-Dimer in serum samples of the (**a**) control beagle and (**b**, **c**, **d**, **e**) AMI beagles (means ± s.d., n = 3).

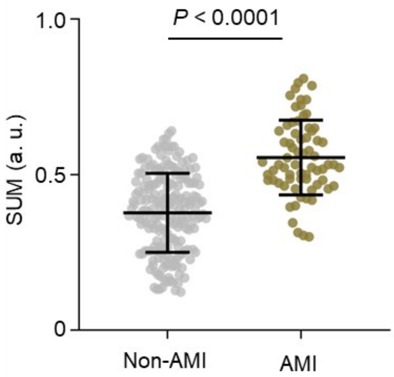

Figure S38. Discriminate AMI patients and non-AMI patients *via* SUM of MIB signatures. SUM values of AMI patients (n = 68) and non-AMI individuals (n = 197) (means ± s.d.). Statistical differences were determined by unpaired two-sided *t*-tests.

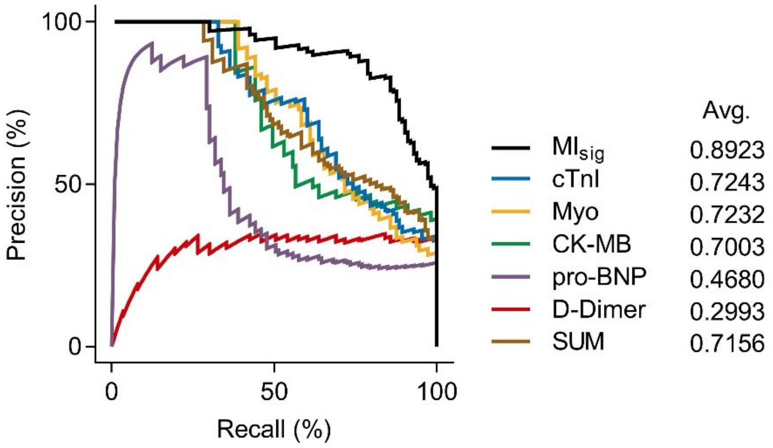

Figure S39. PRC curves for AMI identification. PRC curves and corresponding average precision (Avg.) when using different signatures as criteria to differentiate AMI patients and non-AMI individuals.

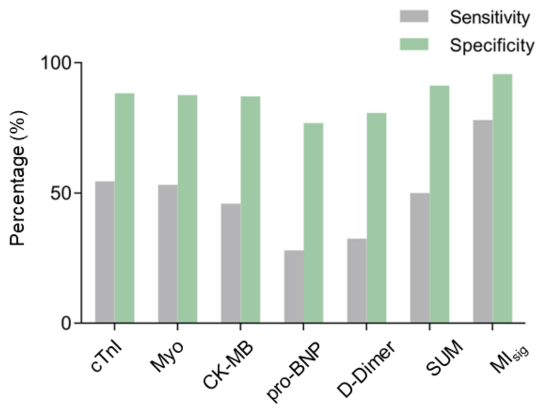

Figure S40. Sensitivity and specificity of AMI diagnosis *via* MIB signatures. Comparison of sensitivity and specificity in AMI diagnosis based on MI_{sig} and single protein biomarkers.

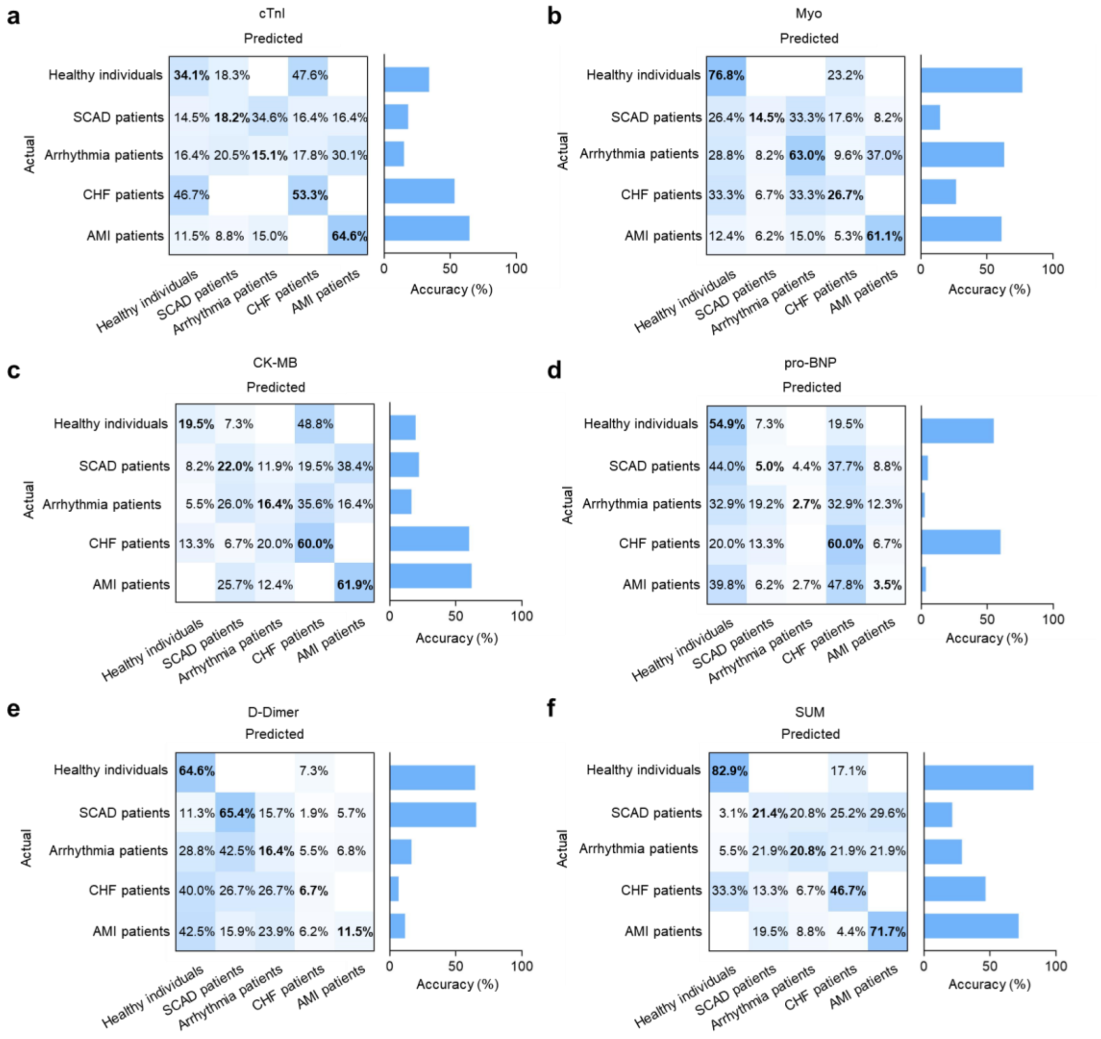

Figure S41. Cardiovascular disease classification results *via* single MIB signatures and SUM. Confusion matrix summarizing the cardiovascular disease classification results based on (**a**) cTnI, (**b**) Myo, (**c**) CK-MB, (**d**) pro-BNP, (**e**) D-Dimer, and (**f**) SUM signatures.

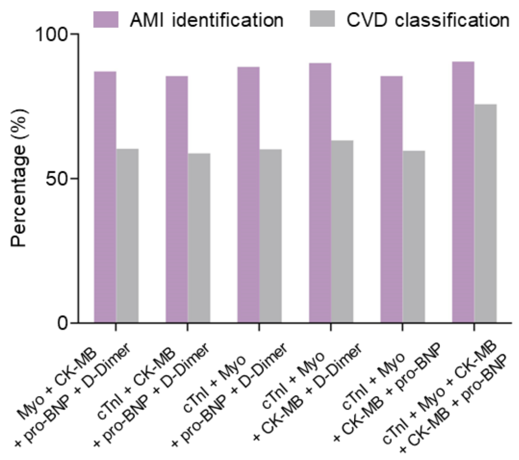

Figure S42. Accuracy of AMI diagnosis and CVD classification *via* different MIB signature combinations. Comparison of identification accuracy in AMI diagnosis and CVD classification based on different MIB signature combinations.

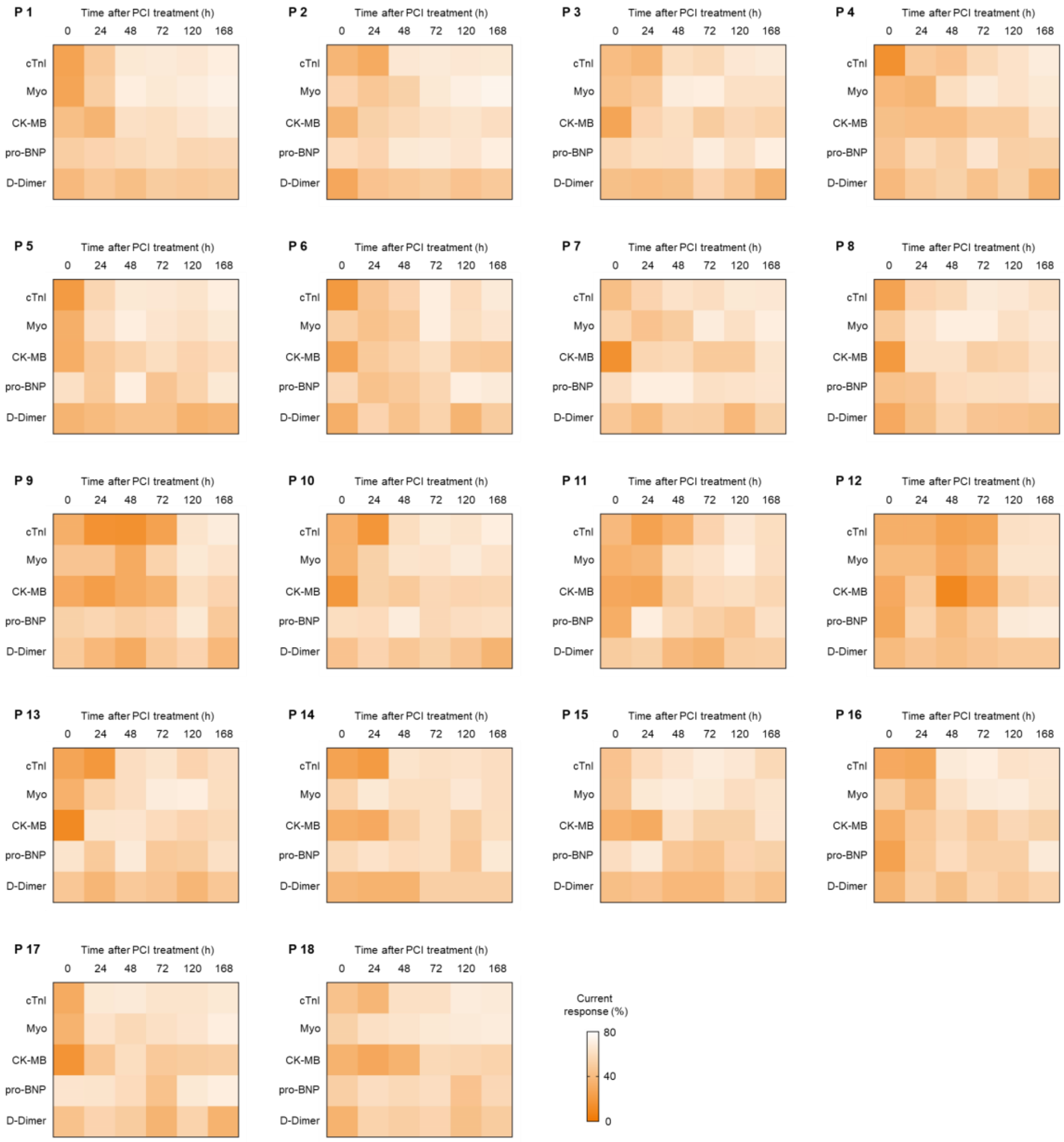

Figure S43. Longitudinal monitoring of 18 AMI patients undergoing PCI treatment. MIB profiles of each patient were obtained within 168 h after PCI treatment. The color intensity indicates the level of MIB signatures.

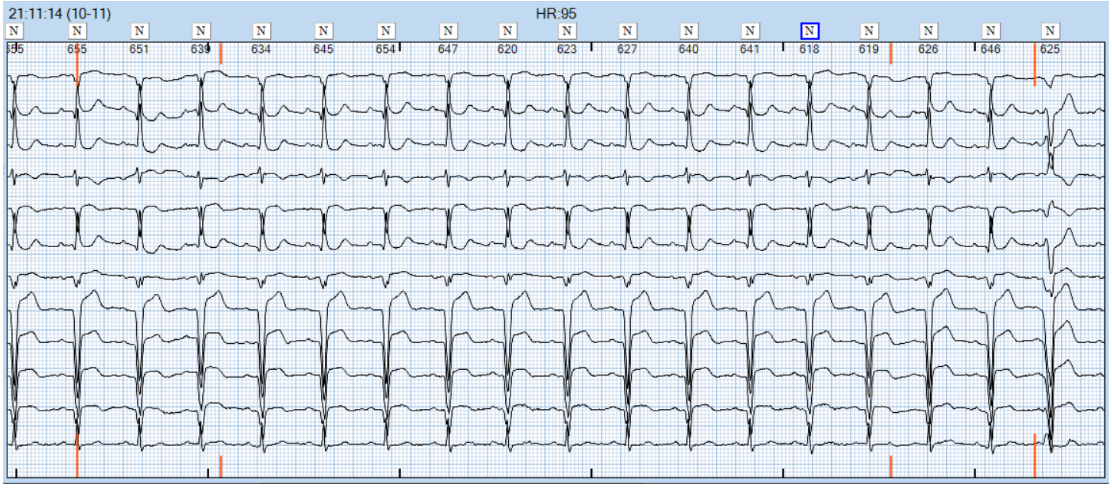

Figure S44. Clinical examination results of patients with poor prognosis. ECG results of patients with suspected poor prognosis 72 hours after PCI. ST-segment changes were observed in the ECG.

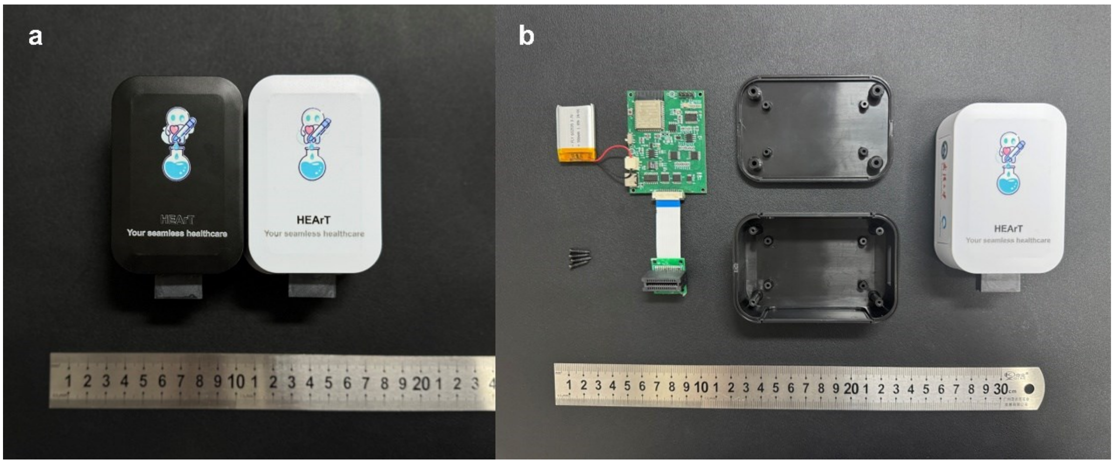

Figure S45. Photos of the HEArT POC devices and their internal structure. (**a**) The appearance, size, and (**b**) internal composition of the HEArT POC device, including the power module, PCB integrated circuit, and array interface.

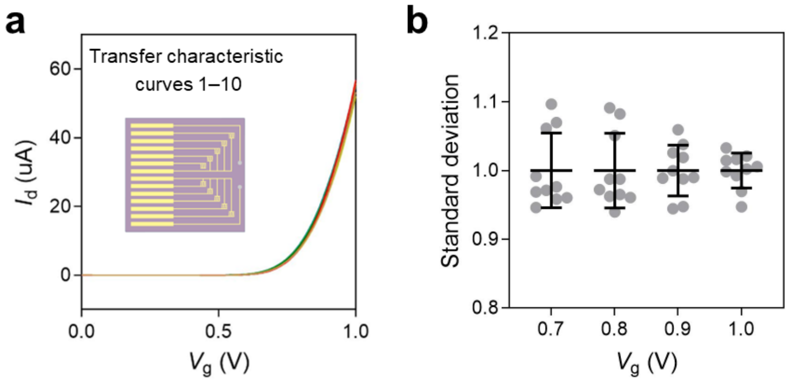

Figure S46. The repeatability of the HEArT POC array. (**a**) The transfer characteristic curves and (**b**) standard deviation of the 10-channel HEArT POC array. The maximum standard deviation is within 10%.

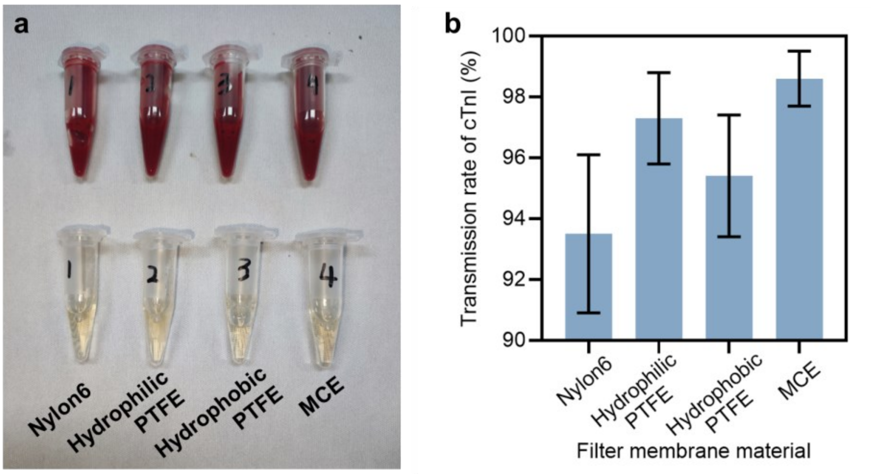

Figure S47. Verification and optimization of filtration performance for serum extraction. (**a**) The serum filtration performance and (**b**) protein permeability of four types of filter membrane materials, including nylon6, hydrophilic PTFE, hydrophobic PTFE, and MCE.

Supplementary tables

Table S1. The detection performance of the MPI FET biosensor toward cTnI, Myo, CK-MB, pro-BNP, and D-Dimer in serum.

Protein	LOD	HLOQ	LLOQ
cTnI	4.2×10^{-12} g/mL	10^{-6} g/mL	10^{-11} g/mL
Myo	7.8×10^{-13} g/mL	10^{-7} g/mL	10^{-12} g/mL
CK-MB	6.3×10^{-12} g/mL	10^{-6} g/mL	10^{-11} g/mL
pro-BNP	5.2×10^{-11} g/mL	10^{-5} g/mL	10^{-10} g/mL
D-Dimer	3.1×10^{-10} g/mL	10^{-5} g/mL	10^{-9} g/mL

Table S2. Summary of the weight ratios and coefficient of variation through 5-fold cross-validation in the training set (n = 20, means ± s.d.).

	cTnI	Myo	CK-MB	pro-BNP	D-Dimer
Healthy individuals	17.5 ± 1.3	21.6 ± 1.5	19.0 ± 0.8	7.7 ± 0.4	33.9 ± 2.3
AMI patients	21.1 ± 1.6	20.5 ± 1.2	32.3 ± 2.2	5.0 ± 0.2	22.2 ± 1.5
SCHD patients	6.7 ± 0.5	16.9 ± 1.4	6.4 ± 0.4	10.0 ± 0.6	59.6 ± 4.4
arrhythmia patients	5.7 ± 0.3	27.5 ± 2.1	40.8 ± 2.8	9.2 ± 0.3	11.4 ± 0.8
CHF patients	22.8 ± 1.8	2.7 ± 0.3	27.9 ± 1.2	30.8 ± 1.6	16.6 ± 1.3

Table S3. Recovery of HEArT biosensing array.

Protein	Protein spiked	Protein detected			Recovery
cTnI	1.00×10^{-10} g/mL	1.15×10^{-10} g/mL	1.10×10^{-10} g/mL	0.95×10^{-10} g/mL	106.7%
	1.00×10^{-9} g/mL	0.86×10^{-9} g/mL	1.06×10^{-9} g/mL	1.23×10^{-9} g/mL	105.0%
	1.00×10^{-8} g/mL	1.15×10^{-8} g/mL	0.94×10^{-8} g/mL	0.88×10^{-8} g/mL	99.0%
Myo	1.00×10^{-10} g/mL	1.23×10^{-10} g/mL	0.96×10^{-10} g/mL	0.92×10^{-10} g/mL	103.7%
	1.00×10^{-9} g/mL	1.35×10^{-9} g/mL	1.12×10^{-9} g/mL	0.95×10^{-9} g/mL	114.0%
	1.00×10^{-8} g/mL	1.22×10^{-8} g/mL	1.15×10^{-8} g/mL	1.06×10^{-8} g/mL	114.3%
CK-MB	1.00×10^{-10} g/mL	0.79×10^{-10} g/mL	0.96×10^{-10} g/mL	1.03×10^{-10} g/mL	92.7%
	1.00×10^{-9} g/mL	1.15×10^{-9} g/mL	0.84×10^{-9} g/mL	0.90×10^{-9} g/mL	96.3%
	1.00×10^{-8} g/mL	1.23×10^{-8} g/mL	1.08×10^{-8} g/mL	1.12×10^{-8} g/mL	113.7%
pro-BNP	1.00×10^{-9} g/mL	0.76×10^{-9} g/mL	0.82×10^{-9} g/mL	0.94×10^{-9} g/mL	84.0%
	1.00×10^{-8} g/mL	1.02×10^{-8} g/mL	0.92×10^{-8} g/mL	1.13×10^{-8} g/mL	102.3%
	1.00×10^{-7} g/mL	0.95×10^{-7} g/mL	0.87×10^{-7} g/mL	1.06×10^{-7} g/mL	96.0%
D-Dimer	1.00×10^{-8} g/mL	1.31×10^{-8} g/mL	0.86×10^{-8} g/mL	0.96×10^{-8} g/mL	104.3%
	1.00×10^{-7} g/mL	0.83×10^{-7} g/mL	0.94×10^{-7} g/mL	0.89×10^{-7} g/mL	88.7%
	1.00×10^{-6} g/mL	1.23×10^{-6} g/mL	1.15×10^{-6} g/mL	1.06×10^{-6} g/mL	114.7%

Table S4. Comparison of the sensing performance of reported POC devices.

Detection method	Probe	Target	Sample or medium	Volume of clinic sample	LoD	Time cost	Clinical validation	Ref.
FET	Molecular electromechanical system	ORF1ab gene	Nasopharyngeal swabs	80 μL	0.01~0.02 copies/μL	0.1~4 min	Yes	1
FET	Absorbed gefitinib	EGFR	Blood samples	10 μL	5.74 fg/mL	N.A.	Yes	2
FET	CRISPR/Cas12a system	Syndrome coronavirus 2	Pharyngeal swab samples	N.A.	40 pM	55 min	Yes	3
FL	CRISPR-Cas12a	*Porphyromonas gingivalis*	Saliva samples	N.A.	12.5 pg/μL	40 min	Yes	4
SWV	Aptamer	HER-2	Human serum samples	N.A.	0.008 ng/mL	N.A.	No	5
FL	Organogel particles	Urinary hyaluronidases	Urine samples	N.A.	~6.44 pM	N.A.	Yes	6
Pt nanoparticle	Antibodies	HCV antigen	Plasma sample	50 μL	3000~ 10000 IU/ml	~23 min	Yes	7

catalysis								
SERS	Antibodies	Cytokeratin-18	Serum samples	N.A.	pg/mL range	N.A.	Yes	8
FET	Antibodies	MIBs	Blood samples	10 µL	pg/ml levels	15 min	Yes	This work

N.A.: not available; **FET**: field-effect transistor; **FL**: fluorescence; **SWV**: square wave voltammetry; **SERS**: surface-enhanced Raman scattering.

[1] L. Wang, X. Wang, Y. Wu, M. Guo, C. Gu, C. Dai, D. Kong, Y. Wang, C. Zhang, D. Qu, C. Fan, Y. Xie, Z. Zhu, Y. Liu, D. Wei, *Nat. Biomed. Eng.* **2022**, *6*, 276-285.

[2] Z. Jiang, D. Ye, L. Xiang, Z. He, X. Dai, J. Yang, Q. Xiong, Y. Ma, D. Zhi, Y. Zou, Q. Peng, S. Wang, J. Li, F. Zhang, C.-a. Di, *Nat. Mater.* **2024**, *23*, 1547-1555.

[3] J. Chen, D. Yang, D. Ji, B. Guo, Y. Guo, H. Lin, R. Zhang, Z. Chang, Y. Lu, G. Zhu, L. Zhao, T. Rungrotmongkol, X. Lu, Q. Ren, W. Wu, Y. Zhang, Y. Fang, *Adv. Funct. Mater.* **2025**, *35*, 2420701

[4] J. Wang, J. Zhang, X. Qu, B. Liu, N. Song, Y. Liu, W. Wang, M. Ding, L. Wang, J. Zhang, Y. Yuan, Q. Ma, L. Tian, Y. Niu, J. Wang, L. Shen, *Chem. Eng. J.* **2025**, *510*, 161578.

[5] S. Rauf, A. A. Lahcen, A. Aljedaibi, T. Beduk, J. Ilton de Oliveira Filho, K. N. Salama, *Biosens. Bioelectron.* **2021**, *180*, 113116.

[6] C. Keum, H. Yeom, T. I. Noh, S. Y. Yi, S. Jin, C. Kim, J. S. Shim, S. G. Yoon, H. Kim, K. H. Lee, S. H. Kang, Y. Jeong, *Nat. Biomed. Eng.* **2025**, *9*, 1026-1038.

[7] H. Chen, Y. Gao, G. Li, M. Alam, S. Udayakumar, Q. N. Mateen, S. Rostamian, K. Cilley, S. Kim, G. Cho, J. Gwak, Y. Song, J. M. Hardie, M. K. Kanakasabapathy, H. Kandula, P. Thirumalaraju, Y. Song, A. Parandakh, A. Bigdeli, G. P. Fricker, J. Gustafson, R. T. Chung, J. Mera, H. Shafiee, *Sci. Adv.*, *11*, eadt3803.

[8] S. Sloan-Dennison, K. M. Scullion, B. Clark, P. Fineran, J. Mair, S. Laing, N. C. Shand, C. Rathmell, D. Creasey, D. Bingemann, J. Faircloth, M. Zieg, E. Varghese, C. J. Weir, J. W. Dear, K. Faulds, D. Graham, *Nat. Commun.* **2025**, *16*, 6223.

Table S5. Summary and Comparison of Electrode Surface Modification Strategies and Detection Performance.

Detection method	Interfacial architecture	Target	LoD	Clinical validation	Ref.
SWV	Agarose gel coating	Kanamycin	20 uM	No	1
FET	Supported lipid bilayers	Avidin	100 pM	No	2
FET	polyethylene glycol (PEG) modification	Prostate-specific antigen	/	No	3
DPV	Zwitterionic peptide	Cortisol	3.5 pg/ml	Yes	4
Chronoamperometry	Zwitterionic poly(sulfobetaine-3,4-ethylenedioxythiophene)	Glucose	/	No	5
FET	Polydopamine (PDA) and polypyrrole (PPy)	SARS-CoV-2 N Antigen	4.6 fg/ml	Yes	6
FET	MPI functionalization	MIBs	4.2 pg/ml	Yes	This work

FET: field-effect transistor; **SWV**: square wave voltammetry; **DPV**: differential pulse voltammetry;

Reference:

[1] S. Li, J. Dai, M. Zhu, N. Arroyo-Currás, H. Li, et al., *ACS Nano* **2023,** *17*, 18525-18538.

[2] D. Lee, W. H. Jung, S. Lee, E.-S. Yu, T. Lee, et al., *Nat. Commun.* **2021,** *12*, 3741.

[3] N. Gao, W. Zhou, X. Jiang, G. Hong, T.-M. Fu, et al., *Nano Lett.* **2015,** *15*, 2143-2148.

[4] Z. Song, R. Han, K. Yu, X. Luo, *Anal. Chem.* **2025,** *97*, 17849-17856.

[5] H. Wu, C.-J. Lee, H. Wang, Y. Hu, M. Young, et al., *Chem. Sci.* **2018,** *9*, 2540-2546.

[6] Q. Peng, W. Huang, D. Chen, Z. Gao, Y. Yang, et al., *Chin. J. Chem.* **2023,** *41*, 2253-2260.

Table S6. List of proteins used in this work.

Protein	Vendor	Origin	Type
anti-cTnI	Abcam (ab10237)	Mouse	IgG1
cTnI	Abcam (ab283299)	Human	
anti-Myo	Abcam (ab77232)	Rabbit	IgG
Myo	Abcam (ab77876)	Human	
anti-CKMB	Abcam (ab404)	Mouse	IgG1
CKMB	Fitzgerald (J31315577)	Human	
anti-proBNP	Abcam (ab239519)	Mouse	IgG2b
proBNP	Abcam (ab42238)	Human	
anti-D-Dimer	Abcam (ab273891)	Rabbit	IgG
D-Dimer	Abcam (ab168863)	Human	
